# Hedgehog Signaling Acts with the Temporal Cascade to Promote Neuroblast Cell Cycle Exit

**DOI:** 10.1371/journal.pbio.1001494

**Published:** 2013-02-26

**Authors:** Phing Chian Chai, Zhong Liu, William Chia, Yu Cai

**Affiliations:** 1 Temasek Life Sciences Laboratory, National University of Singapore, Singapore; 2 Department of Biological Sciences, National University of Singapore, Singapore; Stanford University, United States of America

## Abstract

In *Drosophila* postembryonic neuroblasts, transition in gene expression programs of a cascade of transcription factors (also known as the temporal series) acts together with the asymmetric division machinery to generate diverse neurons with distinct identities and regulate the end of neuroblast proliferation. However, the underlying mechanism of how this “temporal series” acts during development remains unclear. Here, we show that Hh signaling in the postembryonic brain is temporally regulated; excess (earlier onset of) Hh signaling causes premature neuroblast cell cycle exit and under-proliferation, whereas loss of Hh signaling causes delayed cell cycle exit and excess proliferation. Moreover, the Hh pathway functions downstream of Castor but upstream of Grainyhead, two components of the temporal series, to schedule neuroblast cell cycle exit. Interestingly, *hh* is likely a target of Castor. Hence, Hh signaling provides a link between the temporal series and the asymmetric division machinery in scheduling the end of neurogenesis.

## Abstract 2

During the development of the *Drosophila* nervous system, the developmentally regulated Hedgehog pathway, together with a series of temporal transcription factors, schedules the end of neurogenesis.

## Author Summary

In almost all metazoans, neurons are produced by a group of neural stem cells/progenitors in a precise temporal manner, which is important for generating a functional nervous system. In *Drosophila*, this “timing” mechanism is mainly governed by the sequential switching of transcription factors in neural stem cells called neuroblasts, such that neuronal fate is associated with its birth order. These temporal factors also coordinate the termination of neuroblast division towards the end of neurogenesis. In this study, we show that Hedgehog (Hh) signaling also regulates the division rate of neuroblasts during their proliferative phase at larval stage, as well as the cessation of proliferation at early pupal stage. Excessive Hh signaling causes premature neuroblast cell cycle exit and early termination of neurogenesis, while loss of Hh signaling results in prolonged proliferation of neuroblasts beyond its physiological window. We also find that Hh signaling acts in concert with the temporal transcription factors, and is itself regulated by these factors. We hypothesize that this mode of interaction (temporal transcription factors with developmentally regulated signals like Hh) during neurogenesis could be widely conserved in other organisms.

## Introduction

Both intrinsic and extrinsic mechanisms are deployed during development to generate cellular diversity [1]. Extrinsic mechanisms involve cell-cell communication, while intrinsic mechanisms ensure preferential segregation of cell fate determinants into one of the two daughter cells upon completion of cell division. The latter is well exemplified during *Drosophila* neurogenesis [2]–[5]. *Drosophila* embryonic neuroblasts (NBs) delaminate from the neuroepithelium and these neural stem cells undergo repeated self-renewing asymmetric divisions. Each division generates a larger daughter that retains NB identity and a smaller daughter, ganglion mother cell (GMC), that normally divides one more time to produce two neurons/glia depending on lineage specificity. At the end of embryogenesis, most NBs enter a proliferative quiescent stage and subsequently resume mitotic activity during early larval stages. These larval NBs, like their embryonic counterparts, undergo extensively repeated divisions to self-renew and at the same time produce postmitotic neurons/glia to build a functional nervous system [6],[7].

During NB divisions, cell fate determinants including Numb, Prospero (Pros), and Brain tumor (Brat) are asymmetrically localised onto one side of the NB cortex (referred to as the basal cortex) via two coiled-coil adaptor proteins, Partner of Numb (Pon, the adaptor for Numb) and Miranda (Mira, the adaptor for Pros and Brat), and are subsequently segregated into the small GMC daughter at the end of NB divisions [8]–[18]. The basal localization and segregation of these cell fate determinants into GMCs are controlled by two evolutionarily conserved protein complexes: the Bazooka (Baz, the fly Par-3 homolog)/DmPar6/DaPKC (atypical protein kinase C) complex and the Partner of Inscuteable (Pins)/Gαi complex, which localize on the opposite side of the cortex (referred to as the apical side) and are bridged together to form a larger protein complex via Inscuteable (Insc) [19]–[27]. Pros is a homeodomain-containing transcriptional factor and acts as a binary switch between self-renewal and differentiation during neurogenesis [28]. It suppresses genes required for NB self-renewal; but its activity is also required to activate genes necessary for GMC differentiation. Hence mis-expression of Pros in the NBs leads to their loss via precocious differentiation [29],[30], while in the absence of Pros, GMCs fail to differentiate, express NB markers, and exhibit increased proliferation [28]. Thus, an imperative task of NB asymmetric division is to segregate Pros exclusively into GMCs. In embryonic NBs, Pros and Mira are transiently localized onto the apical cortex during late interphase and early prophase prior to their basal localizations. The localization of Pros and Mira is initiated by the DaPKC-mediated direct phosphorylation on Mira, which results in the displacement of Mira from the apical cortex and subsequently, via an unidentified mechanism, localize onto the basal cortex [31]. Recently, the conserved protein phosphatase (protein phosphatase 4 [PP4]) complex was identified as an essential mediator for the localization of Pros and Mira during both interphase and mitosis [32]. In the absence of PP4 activity, Pros and Mira are mislocalized to the nucleus during interphase and cytoplasm during mitosis. Consistent with a role of Pros in suppressing NB self-renewal genes, PP4 mutant NBs exhibit reduced proliferation.

Repeated segregation of the same sets of cell fate determinants does not fully explain how extensive cellular diversity can be generated from NB lineages. The generation of diverse progeny from a single NB is also regulated by another NB intrinsic mechanism such that each NB will undergo a specific number of divisions in a defined temporal and spatial context to generate a lineage with distinct neuronal or glial fates [33],[34]. During embryonic neurogenesis, this “timing” mechanism (or temporal series/mechanism) is controlled by sequential expression of a series of transcription factors in the NBs: Hunchback (Hb)→Krupple (Kr)→POU homeodomain protein 1/2 (Pdm)→Castor (Cas)→Grainyhead (Grh), although some NB lineages only express a subset of this series [35]–[37]. Grh is the last transcription factor expressed in embryonic NBs and its expression persists in the postembryonic NBs throughout the larval stage, presumably to maintain mitotic activity of the NB [38]–[41]. The linearity and robustness of the temporal series involves an intricate network of transcriptional regulation encompassing additional players, such as Seven-up (Svp) [35],[37],[42],[43]. Temporal series continues during the larval stage with the redeployment of embryonic temporal regulators Cas and Svp to achieve two major transitions in NB lineages: (1) the neuronal identity switch from larger Chinmo^+^Br-C^−^ early-born neurons to smaller Chinmo^−^Br-C^+^ late-born neurons at L2 stage, and (2) termination of NB proliferation (cell cycle exit) at pupal stage, which is concomitant with cytoplasmic localization of Mira and a burst of nuclear Pros during early mitosis [44]. Furthermore, an early burst of Pros is sufficient to trigger cell cycle exit in type I NBs and cessation of neurogenesis in larvae. Therefore nuclear Pros may act as the physiological means for promoting NB cell cycle exit and cessation of neurogenesis [44]. However Cas is transiently expressed during early larval development [44]. It is unclear how this transient Cas expression acts to trigger a later burst of nuclear Pros in pupae to promote cell cycle exit. It is also unclear how the temporal mechanism is coupled with the asymmetric division mechanism to generate a functional nervous system.

The Hedgehog (Hh) pathway is repeatedly deployed throughout animal development to mediate diverse functions [45]. The core machinery of Hh signalling is evolutionarily conserved from flies to humans, although there is clear divergence in its mechanistic details [46]. In general, Hh ligands are synthesized as precursor molecules that undergo autocatalytic cleavage to yield an N-terminal fragment with a cholesterol moiety tethered to its C-terminal end before its secretion from producing cells. The receptor for Hh is a 12-pass transmembrane protein, Patched (Ptc), which, in the absence of Hh, inhibits the activity of a second downstream effector molecule, Smoothened (Smo). Smo is a seven-pass transmembrane protein that bears resemblance to the mammalian G-protein coupled receptor (GPCR) [47],[48]. The ultimate effector of Hh signalling pathway is the transcription factor Cubitus interruptus (Ci), which can act as a full length transcriptional activator, Ci-155, or a proteolytically cleaved transcriptional repressor, Ci-75 [49],[50]. In the absence of Hh ligand, Smo activity is suppressed by Ptc and, consequently, Ci is phosphorylated and is processed into the repressor form, Ci-75. The binding of Hh to its receptor inhibits Ptc and alleviates its inhibition of Smo, resulting in the stabilization and phosphorylation of Smo C-terminal tail [51]–[53], and subsequently stabilization of the Ci activator form, Ci-155. In *Drosophila* embryos, Hh signaling is implicated in the specification of a subset of NBs in a spatial pattern. Furthermore, the Hh pathway also functions to reactivate NBs from their quiescent stage during early larval stages [54]. However, its role, if any, after NB reactivation is unknown.

In this study, we investigate the function of Hh signaling during the development of *Drosophila* postembryonic brain. We show that *hh* expression is temporally regulated in the postembryonic larval brain and Hh signaling promotes NB cell cycle exit in the early pupae, possibly by mediating nuclear localization of Pros. Earlier (excess) activation of Hh signaling results in defective Pros localization, and leads to under-proliferation and premature cell cycle exit, whereas loss of Hh signaling causes delayed NB cell cycle exit and excess proliferation. Hh expression in postembryonic larval brain depends on an earlier pulse of expression of a component of the NB temporal series, Cas. Hh signaling in NBs in turn shuts down the expression of Grh, the terminal component of the NB temporal series required for the mitotic activity of larval NBs. Hence the timely exit of NBs from the cell cycle depends on the intricate interplay between Hh signaling, components of the NB temporal series (*cas*, *grh*) as well as Pros, a component of the NB asymmetric division machinery.

## Results

### Hedgehog Signaling Restricts Proliferation of NBs in the Central Brain

**Figure 1:**
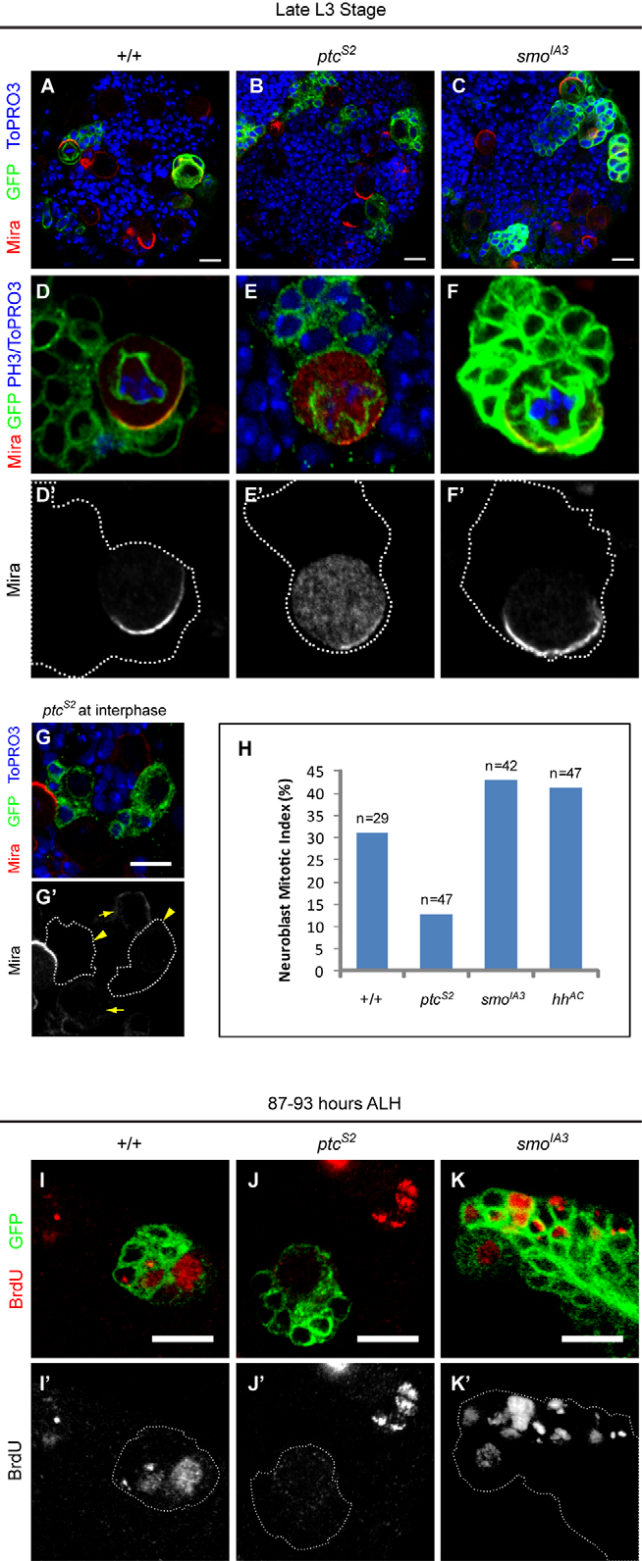
Hedgehog signaling affects the proliferation of NBs. (A–C) Third instar larval brains harbouring *wt* (A), *ptc^S2^* (B), and *smo^IA3^* (C) MARCM clones were immunostained to show the clone size (green, GFP channel), and Mira (red) localization. In *wt* clones (D–D′), and *smo^IA3^* (F–F′) clones, the mitotic NBs showed a strong Mira crescent; but Mira was highly cytoplasmic in *ptc^S2^* NBs (E–E′). In interphase *ptc^S2^* NBs (G–G′, GFP clones), the cortical Mira was weakened or absent (yellow arrowheads) compared to their *wt* counterparts (yellow arrows). DNA was stained with either PH3 (D and F), or ToPRO3 (A–C, E, and G). (H) Quantification of NB mitotic index in *wt*, *ptc^S2^*, *smo^IA3^* and *hh^AC^* clones based on the percentage of the NBs that expressed PH3 at 96 h ALH. (I–K) BrdU (red) incorporation in *wt* (I), *ptc^S2^* (J), and *smo^IA3^* (K) clones labeled by CD8:GFP (green) after 4 h of continuous feeding with BrdU. Scale bar = 10 µm.

**Figure 2:**
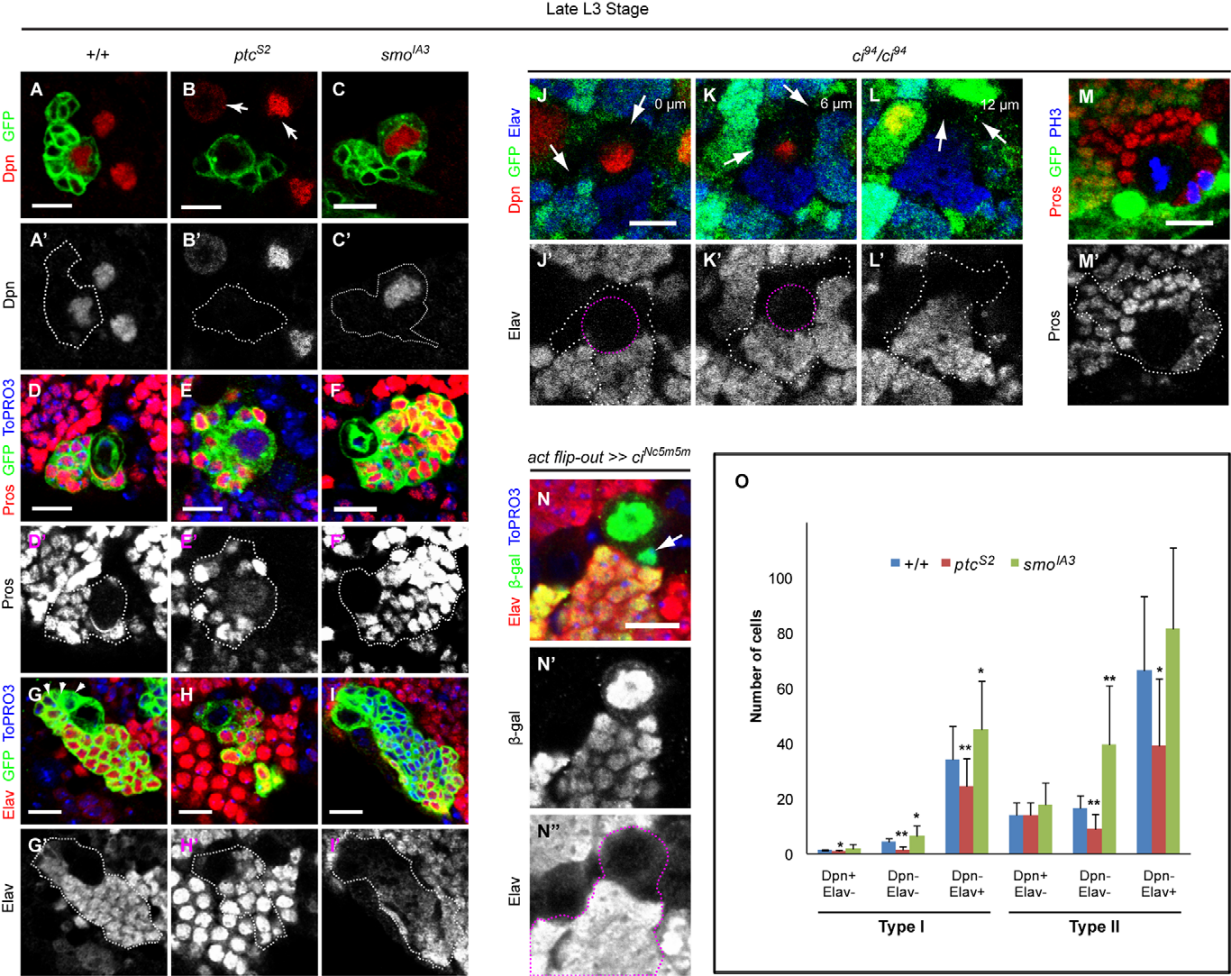
A canonical Hh signaling pathway is required to control NB proliferation but not neuronal differentiation. (A–I′) NB clones of different genotypes: *wt* (A–A′, D–D′, and G–G′), *ptc^S2^* (B–B′, E–E′, and H–H′) and *smo^IA3^* (C–C′, F–F′, and I–I′) were marked by CD8:GFP (green). Dpn was expressed in the nuclei of *wt* NBs (A–A′), and its expression remained unchanged in *smo^IA3^* NB (C–C′). However *ptc^S2^* NB had decreased levels of Dpn expression (B–B′) compared to *wt* NBs located outside of the clone (arrows). *wt* and *smo^IA3^* NBs showed a Pros crescent during mitosis (D–D′ for *wt*; unpublished data for *smo^IA3^*) and did not show visible nuclear Pros expression during interphase (F–F′ for *smo^IA3^*; unpublished data for *wt*), but *ptc^S2^* interphase NBs showed nuclear-localized Pros (E–E′). (G–G′) *wt* NB clone showing three undifferentiated GMCs (arrowheads) which lacked Elav expression. (H–H′) In a *ptc^S2^* NB clone all of the cells surrounding the NB expressed Elav. (I–I′) In *smo^IA3^* NB clones an enlarged cluster of cells surrounding the NB did not express Elav. (J–M′) Homozygous *ci^94^* clones as marked by the absence of GFP. (J–L′) Three consecutive *z*-sections (6 µm apart from each other) of a *ci^94^* clone exhibited areas that are both Dpn and Elav negative, occupied by GMC-like cells (arrows). The pink dotted line marked the outline of the NB (J–K). (M–M′) Pros was localized to the GMCs and neurons of *ci^94^*. (N–N″) Over-expression of constitutively active form of *ci* using *act*-GAL4 flip-out system showed a single undifferentiated GMC (arrow) within the clone. (D–L, N) DNA was stained with ToPRO3. Scale bar = 10 µm. (O) Quantification of three different cell fates based on the absence or presence of Dpn and Elav expressions for both type I and type II NB clones in *wt*, *ptc^S2^*, and *smo^IA3^* backgrounds. Error bars showed standard error of the mean (SEM). Statistical significance was determined using Student's *t* test; *p<0.05; **p<0.003.

Using the mosaic analysis of a repressible marker (MARCM) system [55] to screen for potential signaling pathways required for asymmetric division of type I NBs in the central brain, we found that components of the Hh signaling pathway were involved in regulating aspects of asymmetric division, as well as the proliferative capacity of NBs. To examine the effects of compromised Hh signaling in the central brain, we generated homozygous clones of a mutant allele of *smo*, *smo^IA3^*, which has a substitution mutation in the Cys rich domain of the extracellular N-terminal domain and fails to transduce downstream signaling [56]. Conversely, *hh* gain-of-function clones were produced using a loss-of-function allele, *ptc^S2^*, which fails to repress Smo function and results in constitutive Hh signal activation, even in the absence of the Hh ligand [57]. In wild-type (wt) animals, Baz/Par6/aPKC and Insc/Pins/Gαi proteins form a complex and localize on the apical cortex of dividing NBs (Figure S1A–S1C), and these apical proteins exhibited largely normal localization in *ptc^S2^* and *smo^IA3^* mutant NBs (Figure S1E–S1G, S1I–S1K, and unpublished data). Numb and its adaptor Pon were correctly localized on the basal cortex in all *wt*, *ptc^S2^*, and *smo^IA3^* mutant NBs (Figure S1D, S1H, S1L, and unpublished data). While Mira/Pros complex was localized to the basal cortex in both *wt* and *smo^IA3^* mutant NBs (Figures 1D, 1F, 2D, 2F, S1C, S1K); however, both Mira and Pros were largely delocalized and showed cytoplasmic accumulation in *ptc^S2^* mutant NBs (Figures 1E, 2E, S1G). Similar Mira delocalization defects were seen in another *ptc* allele, *ptc^13^* (Figure S1M). Thus, removing *smo* function from NBs does not cause any noticeable defects on asymmetric division, whereas *ptc^S2^* mutant NBs specifically disrupt the basal localization of the Mira/Pros but not Pon/Numb complex during mitosis.

In addition to these asymmetry defects, we also observed that both *ptc^S2^* and *smo^IA3^* mutant NBs exhibited defective proliferation compared to *wt* NBs. At 96 h after larval hatching (ALH), a typical *wt* type I NB clone induced at 24 h ALH (soon after NB reactivation) contained 40.1±11.7 cells (*n* = 19). However, *ptc^S2^* clones were smaller than their *wt* counterparts and contained 26.9±9.8 cells (*n* = 29, *p* = 0.0001), whereas *smo^IA3^* produced noticeably larger clones with 50.4±20.5 cells (*n* = 24, *p* = 0.0283) (Figures 1A–1F′ and 2O). Moreover, most of the *ptc^S2^* NBs appeared to be arrested in interphase based on the appearance of diffused DNA with markedly reduced cortical Mira (93.3%, *n* = 45), whereas the surrounding heterozygous interphase NBs displayed strong cortical Mira (Figure 1B and 1G). To further confirm that the clone size difference in *ptc^S2^* and *smo^IA3^* mutants was a consequence of alteration in NB proliferative capacity, we measured the mitotic index using phospho-histone H3 (PH3) as a mitotic marker. Indeed, *ptc^S2^* NBs were significantly less mitotically active than their *wt* counterparts, whereas a higher proportion of *smo^IA3^* NBs were engaged in mitosis at all time points examined (Figure 1H). We also noted that the increase in mitotic index in *smo^IA3^* NBs was most likely associated with inactivation of Hh signaling as NB clones mutant for the *hh* null allele, *hh^AC^*, were equally mitotically active (Figure 1H). This phenotype, together with an enlarged *hh^AC^* clone size (49.4±19.3 cells; *n* = 33, *p* = 0.0177; Figure S4A–S4C), indicated that Hh functions in a lineage confined manner to restrict the proliferation of the NBs in the central brain (also see Discussion).

One drawback of using PH3 as a proliferative marker is that its index does not distinguish between alteration in the cell cycle time and proliferative capability of the marked cells. Hence, we conducted a 5-bromo-2′-deoxyuridine (BrdU) labeling assay to investigate the propensity of the NBs to undergo cell cycle progression at around 90 h ALH. By allowing 4 h of BrdU nucleoside analogue incorporation, we found that all the *wt* NBs and 9.0±2.7 of progeny (*n* = 33) had BrdU labeling in the nuclei (Figure 1I). In contrast, about half of the *ptc^S2^* mutant NBs (55.0%, *n* = 34) were devoid of BrdU labeling while most of the rest that managed to incorporate BrdU showed fairly weak signal (Figure 1J). In addition, significantly fewer progeny of *ptc^S2^* mutant NBs (2.7±2.5, *n* = 34, *p*<0.0001) had BrdU labeling compared to their *wt* counterparts. This observation confirms that *ptc^S2^* mutant NBs were not actively proliferating. Conversely, all *smo^IA3^* clones comprised a single BrdU-labeled NB, along with an increased number of progeny that harbored nuclei BrdU (11.4±2.5, *n* = 41, *p* = 0.0001) (Figure 1K), consistent with a higher mitotic index of *smo^IA3^* NBs. Collectively, these data showed that ectopic Hh signal activation resulted in reduction in NB proliferation, while defect in Hh signaling increased the proliferative capability/rate of the NBs.

### Canonical Hh Signaling Controls NB Proliferation but Not Neuronal Differentiation

In order to understand the mechanism that underlies changes in NB proliferation, we sought to examine whether there is any alteration to NB and neuronal fates due to the loss and gain of Hh signaling using molecular markers Deadpan (Dpn), Pros, and Elav. In *wt*, all the NB clones examined contained a single large NB that expresses nuclear Dpn (Figure 2A). The newborn GMC was transiently Dpn-positive due to perdurance of the protein after division. In contrast, Dpn expression was severely reduced in *ptc^S2^* NBs (Figure 2B), while *smo^IA3^* NBs continued to express Dpn as normal (Figure 2C). *smo^IA3^* NB clones, despite having a larger clone size and higher mitotic index than *wt*, did not exhibit supernumerary NB-like cells as seen in *pros* or *brat* mutants [10]. In *wt* and *smo^IA3^* NBs, Pros is under our detection threshold during interphase, but accumulates on the basal side of the NBs during mitosis, while in GMCs and neurons, Pros is nuclear (Figure 2D and 2F). Notably, *ptc^S2^* NBs often exhibited nuclear Pros during interphase and cytoplasmic enrichment of Pros during mitosis (Figure 2E and unpublished data). Since it had been shown recently that Pros-dependent NB cell cycle exit in early pupae may depend on nuclear localization of Pros during interphase [44], our observations suggested that the reduced proliferation in *ptc^S2^* clones may be an indication of premature NB cell cycle exit. Indeed, ectopic *pros* expression during larval stage promoted premature NB cell cycle exit with concomitant down-regulation of NB marker Dpn and delocalization of Mira as observed in *ptc* mutant NBs (Figure S2A and S2B).

To understand if GMC division and neuronal differentiation were affected, we examined the neuronal marker Elav. In *wt* clones, all of the cells expressed Elav, except for the large Dpn-positive NB and three to five Dpn-negative GMCs (4.4±0.9 cells per clone, *n* = 23) adjacent to it (Figure 2G and 2O). In contrast, *ptc^S2^* clones had very few Elav-negative GMC-like cells (1.8±1.2 cells per clone, *n* = 53) compared to the *wt* clones (Figure 2H and 2O). This is consistent with the *ptc^S2^* NBs being less mitotically active. However, *smo^IA3^* clones often contained four to 15 GMC-like cells (6.7±2.6 cells per clone, *n* = 25), which were Elav-negative and in direct contact with, or at close proximity to the NB (Figure 2I and 2O). There were two possible explanations for this GMC-like pool expansion: (1) an increase in NB division rate (which we confirmed with live imaging, see below) leading to accumulation of GMCs or; (2) some GMCs remained mitotically active after division and did not differentiate. Our data favored the first possibility as we failed to detect any GMC clones comprising of more than two cells (*n*>50), suggesting that the GMCs did not undergo extra divisions. Furthermore, differentiation was not arrested in *smo^IA3^* clones as all the mutant cells expressed Elav when examined at adulthood (Figure S3A). These results showed that Hh signaling plays a key role in controlling NB proliferation but does not block differentiation in type I NB lineages. Expectantly, *hh^AC^* clones also contained ectopic GMC-like cells (8.1±1.9 cells, *n* = 12), again reinforcing the view that the diffusion of Hh ligand is restricted within a single lineage (Figure S4A and S4B, see Discussion).

While type I NBs generate GMCs that undergo terminal divisions to produce two neurons/glia, type II NBs generate trans-amplifying GMCs that undergo multiple rounds of asymmetric divisions to generate many neurons/glia [58]–[60]. Hh signaling appears to play a similar role in type II NBs. In *wt*, each type II NB clone contains 96.8±31.1 cells (*n* = 21; Figure 2O). *ptc^S2^* clones were smaller and contained only 61.7±29.9 cells (*n* = 19; Figure 2O), while *smo^IA3^* mutant exhibited larger clones with 138.6±48.6 cells (*n* = 16; Figure 2O). Together, these data suggest that Hh signaling is likely utilized as a common mechanism in regulating NB proliferation in the larval brain.

To assess whether Hh signaling exerts its effect on NBs via the canonical pathway, we examined NB proliferation in *ci* clones. When Ci function was compromised in *ci^94^* mutant [61], NB clones exhibited a GMC-like pool expansion phenotype similar to *smo^IA3^* clones (Figure 2J–2L), albeit at a lower frequency (20.0%, *n* = 15). Pros localization was largely unaffected in both NBs and GMCs/neurons within *ci^94^* clones, similar to those observed in *smo^IA3^* clones (Figure 2M). In contrast, NB clones expressing a constitutively active form of *ci*, *ci^Nc5m5m^*, contained fewer Elav-negative GMC-like cells (2.1±1.1 cells per clone, *n* = 15) similar to *ptc^S2^* clones (Figure 2N). Moreover, ectopic Hh signaling induced by over-expressing a constitutively active form of *ci*, *ci^5m5m^*, or *smo*, *smo^RA1234^*, could also lead to aberrant nuclear Pros localization in the NB, but at a lower phenotypic penetrance than *ptc^S2^* clones (Figure S5 and unpublished data). Incidentally, removing one copy of *smo* in *ptc^S2^* background (*ptc^S2^, smo^3/+^*) largely suppressed NB proliferation defects as these clones typically consisted of 4.1±1.1 GMC-like cells per clone (*n* = 25), a number that was comparable to that in *wt* clones (4.4±0.9 cells; Figure S3B). Furthermore, these NBs exhibited strong Mira crescent (Figure S3C; compared with Figure 1E). Similarly, Pros localization defect was largely rescued as normal crescent could be observed in all mitotic NBs (Figure S3D, compared with Figure 2E). These data indicate that a canonical Hh signaling cascade acts to control proliferation in NB lineages.

### Hh Signaling Promotes Cell Cycle Progression of Postembryonic NBs

**Figure 3:**
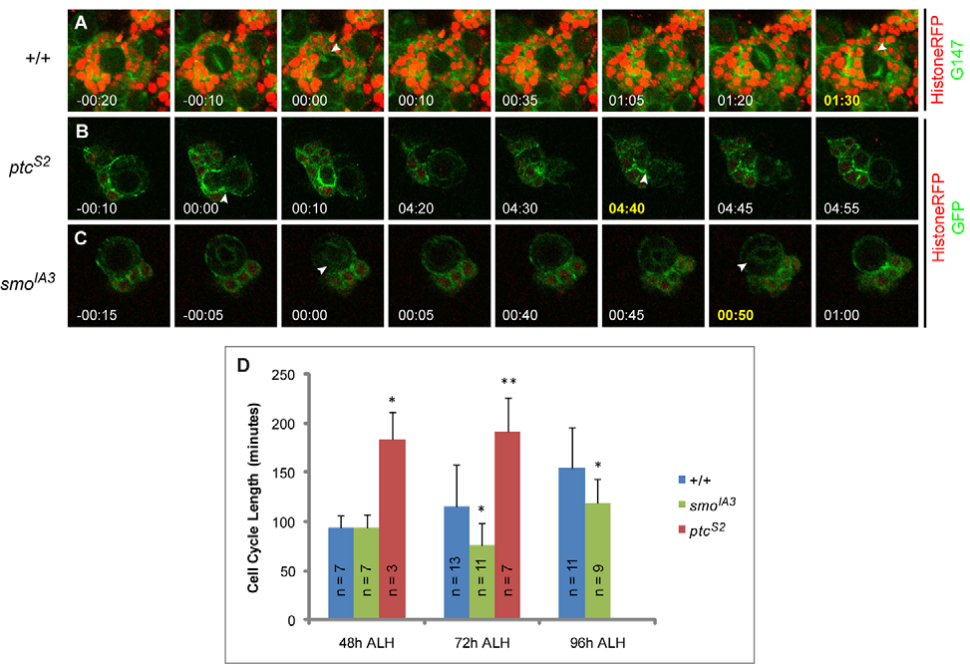
The developmentally regulated cell cycle length of NB is affected by Hh signaling. (A–C) Live-imaging video stills of a 72-h ALH *wt* NB (A) marked by histone-red fluorescent protein (RFP) (red) and G147 (green), as well as those of 72 h ALH *ptc^S2^* (B) and *smo^IA3^* (C) NBs marked by histone-RFP (red) and CD8:GFP (green). Cell cycle lengths were determined by counting the time taken from the appearance of cleavage furrow from one division to the next, on the basis of the appearance of GMC bud (A and B) or nuclear membrane elongation when GMC budded off (C). (D) Quantification of cell cycle length in *wt*, *ptc^S2^*, and *smo^IA3^* NBs, at 48 h, 72 h, and 96 h ALH. *ptc^S2^* NBs failed to divide at 96 h ALH under our live imaging conditions. Error bars represent standard deviation (SD). Statistical significance was determined using Student's *t* test; *p<0.003; **p<0.05.

We next address the direct involvement of Hh signaling in regulating NB division rate by live imaging NBs in various genetic backgrounds. The majority of the postembryonic NBs have a rather tight frame of proliferative window from second instar larval stage ALH till early pupal stage [7]. It has been demonstrated that *Drosophila* postembyonic thoracic NBs exhibit increase in cell cycle time from earlier stage (96 h ALH) to later stage (120 h ALH) [44]. We observed a similar cell cycle trend for the postembryonic central brain NBs as live imaging of *wt* larval brains showed that, under our imaging conditions, their cell cycle lengthened from 93.5±12.8 min at 48 h ALH (*n* = 7) to 115.0±42.8 min at 72 h ALH (*n* = 13), and finally 154.1±42.0 min at 96 h ALH (*n* = 11) (Figure 3A and 3D; Video S1). *smo^IA3^* NBs averaged a cell cycle time that was indistinguishable from that of *wt* NBs at 48 h ALH (93.6±14.1 min, *n* = 7) (Figure 3D). However, the cell cycle lengths of *smo^IA3^* NBs were shorter than their *wt* counterparts at 72 h and 96 h ALH, clocking 76.4±21.8 min (*n* = 11) and 118.9±24.0 min (*n* = 9), respectively (Figure 3C and 3P; Video S2). Conversely, *ptc^S2^* NBs significantly extended their cell cycle length at 48 h and 72 h ALH, averaging 183.3±28.4 min (*n* = 3), and 191.4±34.5 min (*n* = 7) respectively (Figure 3B and 3D; Video S3). In agreement with the low mitotic index at 96 h ALH in *ptc^S2^* NBs, no dividing NB was observed despite many attempts. This implied that most of the NBs had either exited cell cycle (as supported by our BrdU feeding experiments), or had a long cell cycle time that exceeded our technical limitation to keep the explanted brains healthy in culture medium.

**Figure 4:**
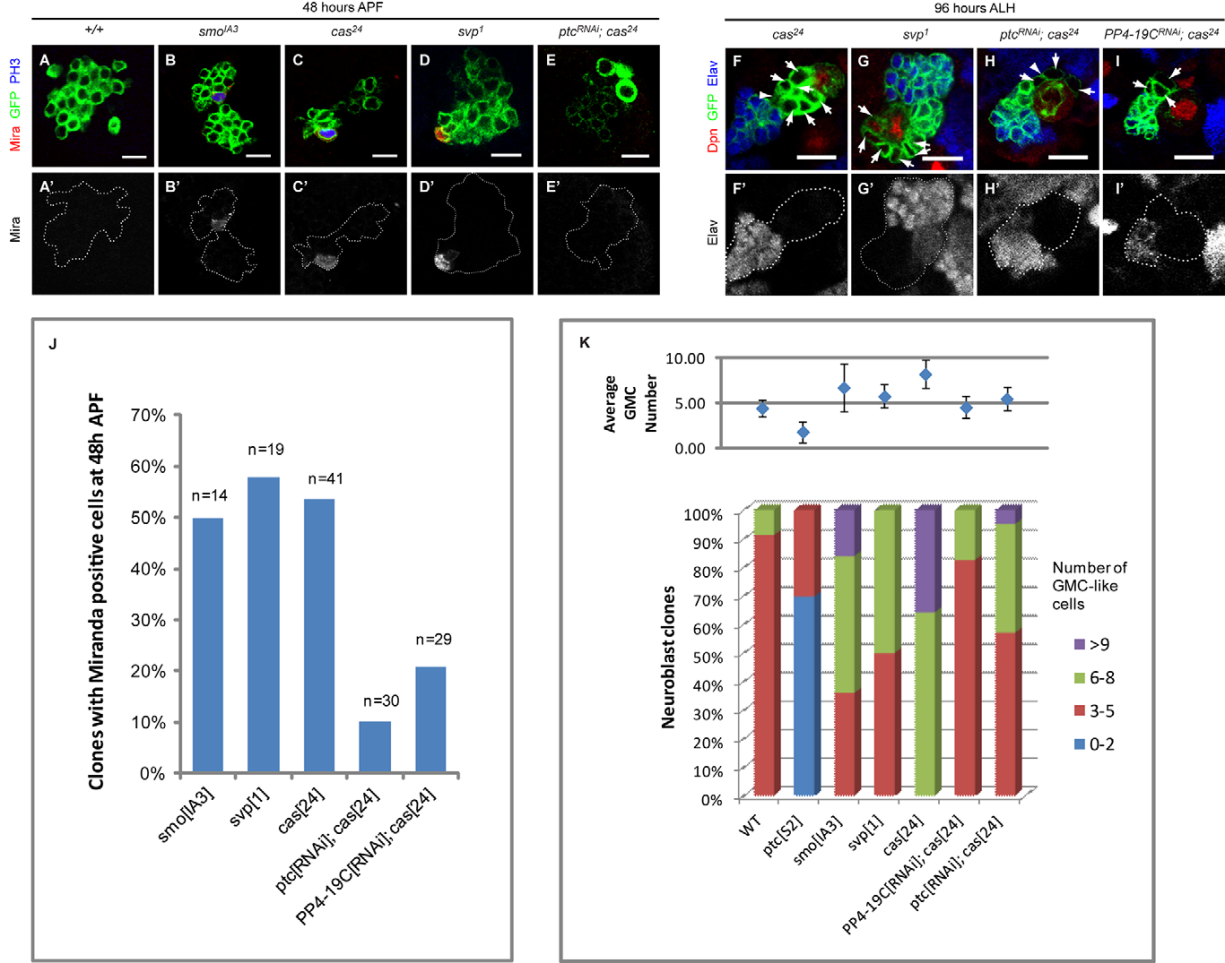
Hh signaling interacts with NB temporal cascade component. (A–E′) About 50% of *smo^IA3^* (B–B′), *cas^24^* (C–C′), and *svp^1^* (D–D′) clones had a single NB that continues to express Mira (red) and PH3 (blue, unpublished data for *svp^1^*) at 48 h APF. Meanwhile, none of the cells within any *wt* clone (A–A′) express Mira or PH3 at that time point. Such delay in cell cycle exit could be largely reverted by expressing *ptc^RNAi^* in the *cas^24^* mutant background (E–E′). (F–G′) *cas^24^* and *svp^1^* clone had increased numbers of Dpn-negative, Elav-negative, GMC-like cells at 96 h ALH (Dpn in red, Elav in blue; arrows, in focus; and arrowhead, out of focal plane). (H–I′) This phenotype of *cas^24^* clones can be largely suppressed by the introduction of *ptc^RNAi^* (H–H′) or *PP4-19C^RNAi^* (I–I′). Note the number of GMC-like cells (arrows and arrowhead). Scale bar = 10 µm. (J) Quantification of 48 h APF clones harboring Mira positive cells in various backgrounds. (K) Quantification of GMC numbers in various mutant backgrounds. A typical *wt* NB clone contained three to five GMC-like cells which are Dpn- and Elav-negative. In *ptc^S2^* clones, there was a decrease in the number of GMC-like cells while *smo^IA3^*, *svp^1^*, and *cas^24^* clones had ectopic GMC-like cells. The number of GMC-like cells in *cas^24^* clones could be reduced to a level close to that of *wt* with the expression of *ptc^RNAi^* or *PP4-19C^RNAi^*.

Studies in mammalian systems have shown that cell cycle lengths of neural precursors increase due to lengthening of the G1 phase as development proceeds [62]. We wondered whether the shorter cycling time in *smo^IA3^* NBs could reflect a “younger state” that has a greater developmental potential. Indeed, 50% of the *smo^IA3^* NBs (*n* = 14) continued to express NB proliferation markers Mira and PH3 at 48 h after puparium formation (APF) (Figure 4B and 4J), when all the *wt* NBs had exited cell cycle according to their normal developmental schedule (Figure 4A) [6],[7],[63]. Along with the proliferative defects seen in *ptc^S2^* NBs, these observations led us to speculate that Hh signaling might have a role in promoting timely postembryonic NB cell cycle exit. It is known that the timing for termination of NB proliferation in the central brain is under the control of the temporal series with Cas and Svp as two essential players [44]. As implicated by their roles in scheduling cell cycle exit, many of *cas^24^* and *svp^1^* clones (53.7%, *n* = 41 and 53.0%, *n* = 34, respectively) retained a single Mira-positive, PH3-positive NB at 48 h APF, in a strikingly similar manner as *smo^IA3^* clones (Figure 4B–4D and 4J). Incidentally, both *cas^24^* and *svp^1^* clones also showed an expansion of GMC-like cells (8.2±1.5 cells and 6.0±1.3, respectively, compared to 4.4±0.9 per clone in *wt*) at 96 h ALH, reminiscent of clones with compromised Hh signaling (Figure 4F and 4G, 4K). These data raise the possibility that Hh signaling may control NB cell cycle exit in conjunction with the temporal series.

Supporting the notion that Hh signaling is likely to exert an effect on NB cell cycle progression, we found that *ptc^S2^* NBs down-regulated cyclin E (CycE) prematurely at 96 h ALH compared to *wt* NBs that retained high level of CycE expression in the nucleus (Figure S7A and S7B). While *smo^IA3^* NBs showed normal CycE expression at 96 h ALH, its expression persisted at 24 h APF, consistent with the fact that most *smo^IA3^* NBs remained mitotically active even at pupal stage (Figure S7C and S7D). Similarly, *cas^24^* clones were also found to harbor CycE expressing NBs at 24 h APF (Figure S7E).

### An Early Transient Pulse of Cas Expression Is Required for the Later *hh* Expression

**Figure 5:**
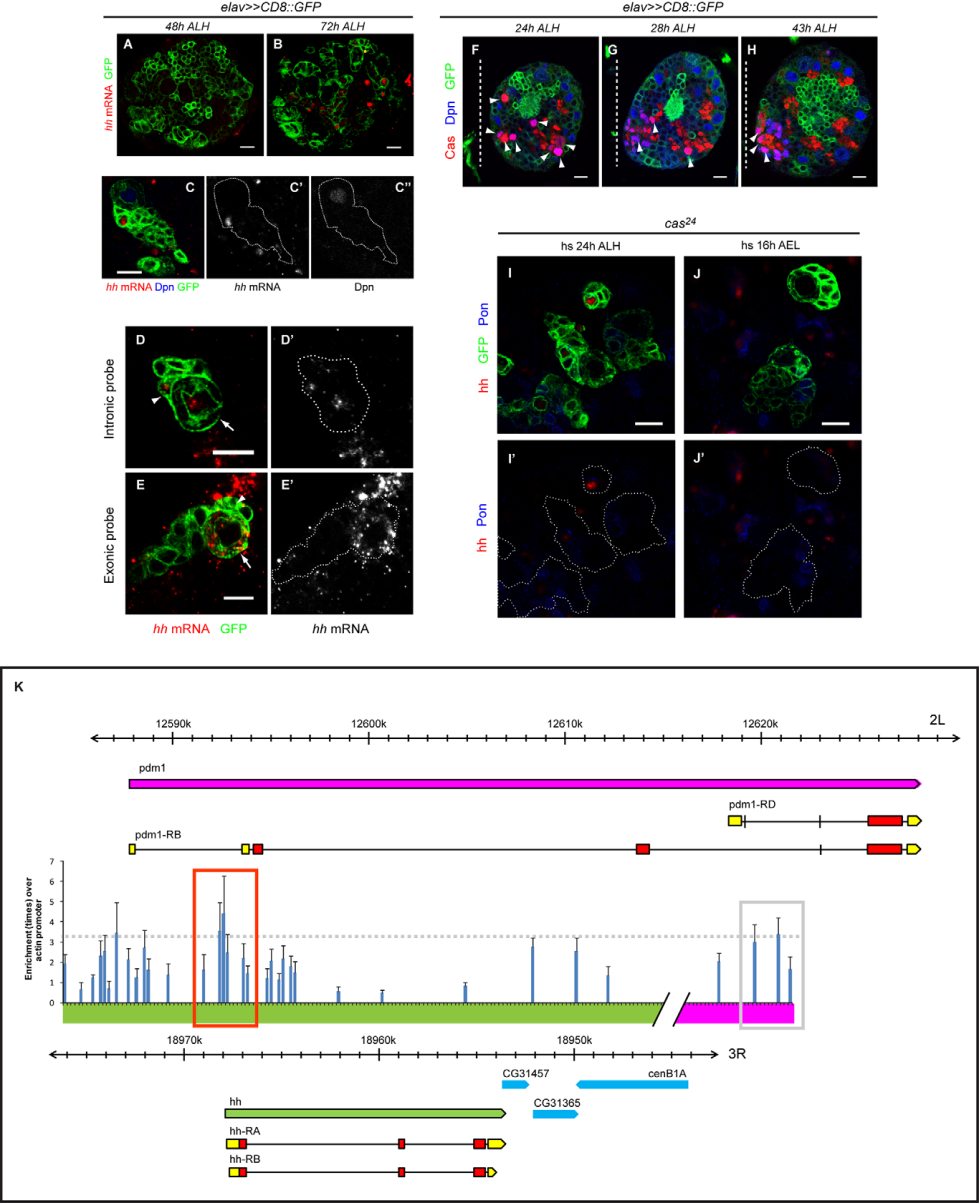
An early transient pulse of Cas is required for later *hh* expression. (A–B) *hh* transcript (red) was detected mainly in the GMCs of 72 h ALH brain lobe (B), but not in that of 48 h ALH brain lobe (A). CD8:GFP (green) was driven with *elav*-Gal4 to mark the outlines of the cells. (C–C″) In situ hybridization of *hh* (red) in a *wt* clone showed that the transcript was expressed in the GMC adjacent to the Dpn-expressing NB. (D–D′) *In situ* hybridization using an intronic probe that detects *hh* pre-mRNA (red) in a *wt* clone showing *hh* expression in the mitotic NB (note the nuclear morphology, arrow), as well as in the GMC next to it (arrowhead). (E–E′) The mature form of *hh* mRNA (red) was detected in the cytoplasm of the NB (arrow) and to a lesser extent, the adjacent GMC (arrowhead). (F–H) Immuno-staining against Cas (red) showed its expression in cells (NBs and intermediate neural precursors [INPs]) that were also co-expressing Dpn (blue, arrowhead), as well as in some other neurons at different developmental time points. The outlines of the cells were marked with membrane GFP (green). The brain lobes were position such that the dorsal side is facing up and the dotted line indicated the midline of the brain. (I–J′) In situ hybridization of *hh* mRNA showed that embryonic Cas was required for normal *hh* expression. *cas^24^* clones induced at 24 h ALH did not affect *hh* expression (I–I′) while *cas^24^* clones induced during late embryonic stage could reduce or abolish *hh* expression (J–J′). Pon (blue) showed the outline of the newly born GMCs that typically expressed *hh* mRNA. (A–F, I–J) Scale bar = 10 µm for all panels. (K) ChIP for Flag-tagged Cas transfected into S2 cells showed that Cas was heavily enriched within the 6-kb region at the 5′ UTR of *hh* gene (orange box). There are 19 putative Cas binding sites within that region. The enrichment of any region of the chromatin was counted as the multiple of specific binding (anti-Flag) against non-specific binding (anti-IgG), and normalized to the enrichment at the actin promoter site (negative control). A known target of Cas, pdm-1 was used as a positive control (grey box) for comparison purposes. The value of the blue bars was the average enrichment (times) from three independent transfections and five independent ChIPs. Error bar correspond to standard error of the mean (SEM).

Is Hh signaling temporally regulated during NB development? Using RNA in situ hybridization against an intronic region of *hh*, which detected nascent nuclear transcripts, we first detected *hh* transcripts in late L2/early L3 larval stages and its expression level strongly increased in late L3 stage brains (Figure 5A and 5B) (see Methods for staging of larvae). Labeling of a single NB lineage showed that *hh* transcripts were expressed in the nuclei of the GMCs, particularly the newborn GMCs, based on their close proximity to the NB (Figure 5C and 5D). Interestingly, the dynamics of *hh* transcripts appeared to be cell-cycle specific in NBs as they were below detection threshold during interphase (Figure 5C), but became detectable during mitosis, suggesting that *hh* is likely transcribed during the G2 phase (Figure 5D). As the intronic probe detects *hh* pre-mRNA and RNA splicing is repressed during M-phase [64], we used an exonic probe to detect the presence of mature *hh* mRNA in interphase NBs. Predictably, mature *hh* cytoplasmic transcripts were detected in NBs and adjacent GMCs, but not in neurons (Figure 5E). Consistent with this expression pattern, Hh protein was found to show a corresponding age-dependent accumulation. Hh was not detected in L2 brains but became progressively apparent by L3 stage (Figure S6A and S6B). Interestingly, Hh protein built up and clustered around the cell surfaces as well as in intracellular puncta of some NBs. Hence, it is likely that Hh acts directly on the NBs to control their proliferation. Moreover, the pattern of *hh* reception using a *ptc* reporter line carrying a 2.8-kb upstream sequence fused to lacZ, as well as a *ptc^H84^* enhancer trap insertion line (Figure S6C and unpublished data), clearly showed that NBs (and possibly the GMCs as well) were the signal receiving cells.

We also investigated the temporal expression profile of *cas* during development and found that Cas was transiently expressed in NBs during early larval development at about 24 h ALH, which coincided with timing of NB reactivation (Figure 5F) [7],[65], and its expression became quickly restricted to a small population of NBs by 28–30 h ALH (Figure 5G). Subsequently, Cas was detected mainly in GMCs and neurons, as well as a cluster of smaller-sized, Dpn-expressing intermediate neural precursors (INPs) located at the dorsal medial region of the central brain (Figure 5H).

Since Cas acts as a transcription factor [66] and given the temporal expression profiles of *hh* and *cas*, we tested the possibility that *hh* expression might be under the control of *cas*. In *wt*, *hh* transcript was often detected in the GMCs, which could be identified by the presence of cortical Pon. Although *hh* transcript levels remain high in the majority of the *cas^24^* mutant clones induced at early L2 after the pulse of Cas during NB reactivation (68.4%, *n* = 19) (Figure 5I), *hh* expression was abolished or significantly reduced in 82.6% of the *cas^24^* clones (*n* = 23) induced during embryonic development, presumably affecting the *cas* expression in early larval stage (Figure 5J); thus placing *hh* downstream of *cas* and suggesting that the competency for *hh* expression was likely dependent on the larval pulse of Cas, which occurred around 24 h ALH (Figure 5F). Intriguingly, constitutive expression of Cas by inducing *UAS-cas* clones in the central brain at both embryonic and larval stages (Figure S8A and S8B) also affected *hh* expression in a similar fashion as *cas* loss-of-function. Together, these data showed that stalling the temporal series either by removing or mis-expressing *cas* could negatively influence *hh* expression. Furthermore, *UAS-cas* expressing clones often harbored a Mira-positive NB at 24 h APF (Figure S8C), signifying that misregulation of temporal progression, similar to loss of *hh* signaling, can also extend the NB proliferative phase beyond its normal developmental limit.

Cas is a zinc finger protein capable of acting both as a transcriptional activator and repressor by binding to recognition sites with the consensus sequence of G/C C C/T C/T AAAAA A/T N [35]. Does the regulation of *hh* expression by Cas reflect the direct binding of Cas to the *hh* promoter? By scanning about 30 kb of genomic sequence flanking *hh* locus, we identified multiple potential DNA-binding sites that contained the Cas recognition sequence. We performed quantitative chromatin immunoprecipitation (ChIP) assays on S2 cells transiently transfected with Flag-tagged Cas and used the genomic region from *pdm-1*, a known Cas target, as a positive control. Cas directly binds to several consensus sites in the genomic region flanking *pdm-1* ([35] and personal communication with W. Odenwald). Indeed, ChIP results showed that the *pdm-1* cis-regulatory region were about three times more enriched with Flag-tagged Cas compared to control *actin-5C* promoter site (Figure 5K) and it was approximately 18 times more enriched compared to the non-transfected control (unpublished data). Supporting the notion that Cas directly associates with *hh* genomic region, Cas was highly enriched at a 6-kb genomic region encompassing *hh* transcription initiation site where there were 19 sites that matched at least eight out of ten bases of the Cas consensus binding sequence (Figure 5K). Specifically, the enrichments for Cas at *hh* genomic region compared to that at *actin-5C* promoter site and to the non-transfected control were up to 4.2 and 21.6 times, respectively. Thus, our results suggest that Cas is a direct positive regulator of *hh*, and its transient pulse of expression during early larval development is necessary for the subsequent Hh expression in postembryonic GMCs.

To further substantiate the relationship between *cas* and *hh* expression, we examined whether Hh signaling interacts genetically with *cas*. As shown earlier, *cas^24^* NBs underwent persistent proliferation till late pupal stage as evidenced by the expression of proliferative markers Mira and PH3 at 48 h APF (Figure 4C and 4J). Ectopic activation of Hh signaling in this background by simultaneous expression of *ptc^RNAi^* partially suppressed the extended proliferation phenotypes (persistent Mira expressing NBs; Figure 4C, 4E, and 4J) as well as expanded GMC-like cells (Figure 4F, 4H, and 4K), while NBs expressing *ptc^RNAi^* alone did not exhibit any noticeable phenotype compared to *wt* NBs at that stage (unpublished data). These data indicate a clear genetic interaction between Hh signaling and *Cas* (thus the temporal series) (Figure 4J and 4K). To further substantiate this interaction, we used an alternative approach to introduce elevated Hh signaling in *cas^24^* clones. It has been shown that the PP4 and PP4R subunits complex functions to down-regulate Hh signaling by dephosphorylating and destabilizing Smo [67]. Hence, compromising PP4 activity leads to elevated Hh signaling activity. Indeed, suppression of the persistent proliferation phenotype associated with *cas* mutant NBs was also evident when PP4-19C, the catalytic subunit of PP4 complex, was compromised in the *cas^24^* background (Figure 4I–4K). The total GMC-like cells within *cas^24^* clones were brought down from 8.2±1.5 (*n* = 14) to 5.5±1.3 (*n* = 23) and 4.5±1.2 (*n* = 21) cells per clone with the introduction of *ptc^RNAi^* and *PP4-19C^RNAi^* transgenes, respectively, a level close to that seen in *wt* clones (Figure 4K). Similarly, unlike *cas^24^*, the majority of the NBs in *ptc^RNAi^; cas^24^* and *PP4-19C^RNAi^*; *cas^24^* double-mutant clones were no longer mitotically active at 48 h APF (Figure 4E and 4J). Together, these data indicate that Hh signaling interacts genetically with the temporal series.

Previous studies placed *svp* downstream of *cas* in type I NB lineages during the progression of temporal series at larval stage [44]. Incidentally, we found that *svp* clones exhibited GMC-like cell expansion as well as an extension of the NB proliferation window similar to those seen in *cas* mutants (Figure 4D, 4G, 4J, and 4K). As Cas is potentially a direct regulator of Hh, we first sought to investigate if Hh signaling could in turn, affect the expression of *svp*. In *wt* clones, Svp was detected in the nucleus of the NBs and a minority of neurons at 40 h ALH (Figure S9A). Interestingly, *svp* expression was unaffected in *smo^IA3^* clones in which Hh signaling was compromised (Figure S9C), indicating that Hh signaling does not function upstream of *svp*. Consistent with this observation, neither did up-regulation of Hh signaling in *ptc^S2^* clones cause ectopic (or elevated) *svp* expression (Figure S9B). It is also conceivable that there is a requirement for *svp* as a downstream component of *cas* to activate *hh* expression. Surprisingly, *hh* transcript remained detectable in the GMCs of *svp^1^* mutant clones, whereas constitutive *svp* mis-expression clones did not trigger ectopic *hh* expression (Figure S9D and S9E). These results indicated an unlikely placement of *hh* downstream of *svp* as well. To gain a better perspective of Svp in the temporal series, we looked at its temporal expression pattern in the postembryonic central brain. We found that *svp* was expressed at a high level in the NBs at 24 h ALH, as early as the time when *cas* expression was detected (Figure S9F). However, unlike *cas*, which was expressed in a short pulse, *svp* had a much wider expression window where medium levels of Svp can be detected in the NBs until 50 h ALH and it continued to be expressed weakly till 96 h ALH (Figure S9G–S9I and S9K). Given the similar time frames of *cas* and *svp* expression at early larval stage, we generated embryonic *cas^24^* clones and assayed for *svp* expression at 24 h ALH. It was found that *svp* expression was not abolished in *cas* mutant NBs, showing that *svp* might not be genetically downstream of *cas* in the NBs of the central brain (Figure S9J).

### Hh Signaling Interacts with Grainyhead to Orchestrate NB Cell Cycle Exit

How does Hh signaling act to control NB cell cycle exit downstream of Cas? One of the positive targets of the temporal series is Grh, which is expressed as a terminal temporal series component in the late embryo. It is expressed upon activation by Cas and its expression persists during larval stages to maintain the mitotic activity of type I NBs [36],[38],[40],[44]. Down-regulation of Grh in the NBs coincides with cell cycle exit, which is accompanied by the reduction of NB size, delocalization of Mira from the cortex to the cytoplasm during early mitosis, as well as Pros translocation into the nucleus [44]. We noted that *ptc^S2^* NBs also exhibited similar Mira and Pros mislocalization (Figures 1E and 2E), although there were rare events in which some *ptc^S2^* NBs managed to reach telophase with poorly localized Mira and Pros along the entire mitotic phase (Figure S10A–S10D). Moreover, there was a noticeable enrichment of Pros within the nuclei of interphase *ptc^S2^* NBs as compared to the neighbouring *wt* NBs with 22.8%±4.1% increase in the intensity of Pros (*n* = 72, *p*<0.0001) (Figures 2E, S10A, and S10E). In addition, the size of *ptc* mutant NBs at 96 h ALH (9.9±1.2 µm, *n* = 72 for *ptc^S2^* mutant, and 9.2±1.1 µm, *n* = 36 for *ptc^13^* mutant) was consistently smaller than that of *wt* NBs (11.2±0.9 µm, *n* = 61) (*p* = 1.0×10^−10^) (Figure S11A–S11C). We hypothesized that Hh signaling might function through Grh in promoting NB cell cycle exit, hence we examined the pattern of Grh expression in NB clones at various time intervals from early L3 until 24 h post-pupation.

**Figure 6:**
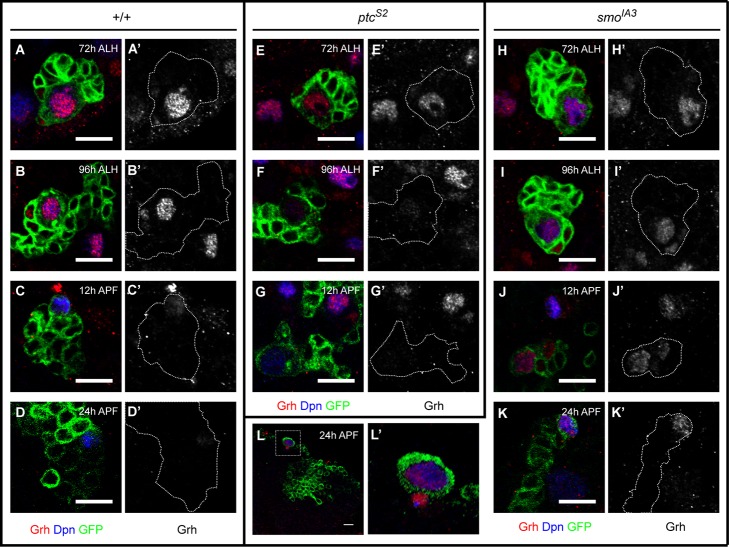
Hh signaling affects the maintenance of *grh* expression. (A–D′) Grh (red) was expressed in *wt* NBs (marked by Dpn, blue) and some GMCs at 72 h ALH (A–A′) and 96 h ALH (B–B′). Grh expression was down regulated in the NBs and abolished in the GMCs by 12 h APF (C–C′) and its expression was barely visible at 24 h APF (D–D′). (E–G′) *ptc^S2^* NB showed normal expression of Grh at 72 h ALH (E–E′) but its level quickly decreased by 96 h ALH (F–F′) and was completely abolished at 12 h APF (G–G′). (H–L′) Grh expression was detected in *smo^IA3^* NB and GMCs at 72 h ALH (H–H′), and 96 h ALH (I–I′) but persisted until 12 h APF (J–J′) and failed to be down-regulated at 24 h APF (K–L′). (L) A *smo^IA3^* NB at 24 h APF with persistent Grh expression amidst the wt background (non-green) in which all the NBs had down-regulated their Grh expression. Scale bar = 10 µm.

In *wt* L3, within the window of 72–96 h ALH, a high level of Grh was always detected in the NBs in conjunction with relatively lower expression levels in some GMCs (Figure 6A and 6B). Down-regulation of Grh occurred at around 12 h APF during which the NBs retained low levels of Grh while the GMCs were devoid of Grh (Figure 6C). By 24 h APF, Grh became barely visible in the NBs (Figure 6D), consistent with the reported cessation of NB proliferation between 20–30 h APF [7]. However, in *ptc^S2^* clones, NBs appeared to down-regulate Grh expression prematurely. At 72 h ALH, Grh expression was absence from GMCs and its level was significantly reduced in NBs at 96 h ALH, and subsequently lost completely from all NBs at 12 h APF (Figure 6E–6G). This was consistent with the lack of mitotic activity in *ptc^S2^* NBs (Figure 1H), presumably most of the NBs had exited the cell cycle (or struggled with cell cycle progression) by 96 h ALH. As for *smo^IA3^* clones, the NBs and some GMCs persistently expressed Grh from larval stages until 12 h into pupal stage (Figure 6H–6J). At 24 h APF, all NBs (*n* = 8) within *smo^IA3^* clones remained Grh-positive but some GMCs began to down-regulate Grh expression (Figure 6K and 6L). More intriguingly, Grh expression persisted in all of the NBs for all the *smo^IA3^* clones examined at 36 h APF (*n* = 16), and as much as 86.7% of the clones still retained some level of Grh expression in the GMCs (Figure S11D). The prolonged expression of Grh in the *smo^IA3^* NBs could probably explain the presence of NB proliferative markers Mira and PH3 in some of the *smo^IA3^* clones at 48 h APF (Figure 4B and 4J). Similar observation was obtained for *ci^94^* homozygous clones in which 90.1% of the Dpn-positive NBs retained Grh expression, and up to 45.5% of these NBs were surrounded by Grh-expressing GMCs at 24 h APF (*n* = 22) (Figure S11E).

**Figure 7:**
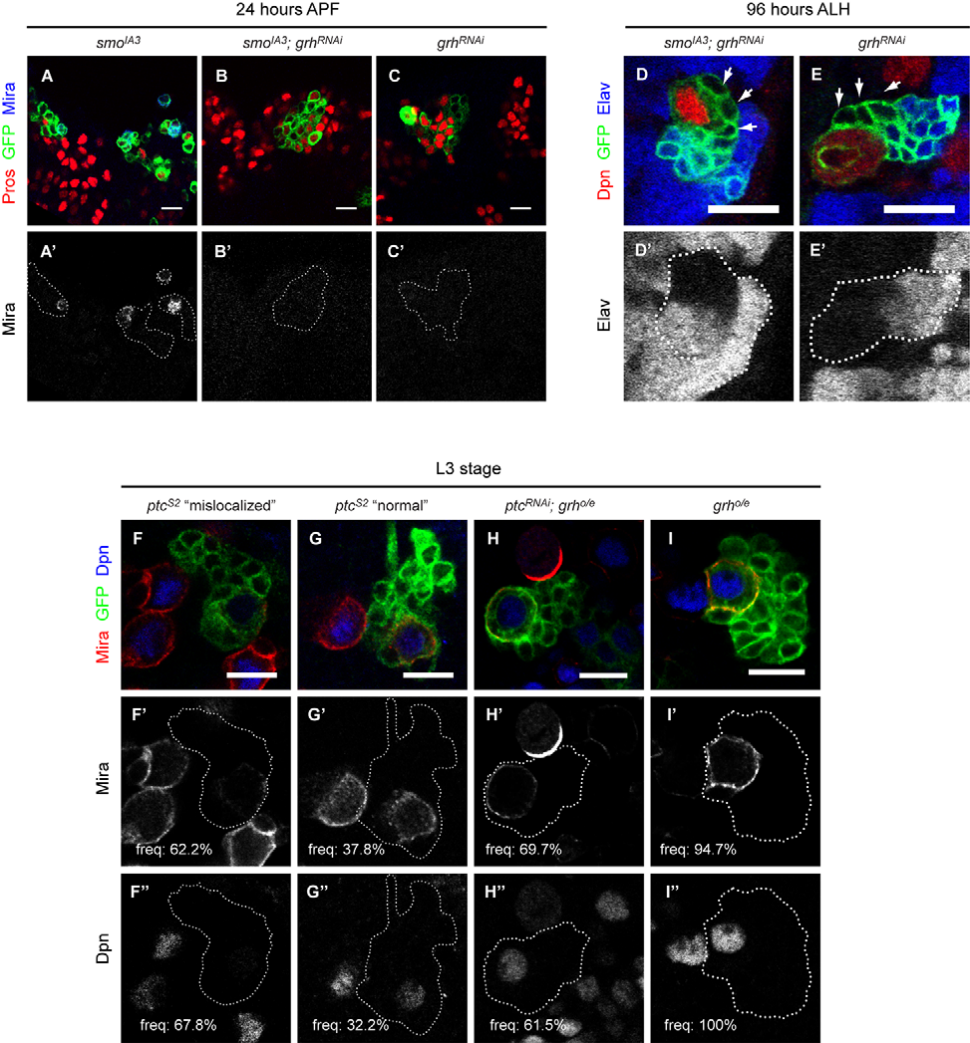
Hh signaling interacts genetically with *grh* to orchestrate NB cell cycle exit. (A–C′) *smo^IA3^* clones (A–A′) marked with CD8:GFP (green) often contained a single NB that expressed Mira (blue) but was devoid of nuclear Pros (red) at 24 h APF. However, no Mira expressing cell could be found in *smo^IA3^* clone that had Grh level reduced by RNA interference (B–B′). As a control, clones expressing *grh^RNAi^* transgene (C–C′) alone did not contain any Mira expressing cell either. RNA mediated knock-down of *grh* in *smo^IA3^* background at 96 h ALH (D–D′) significantly rescued the ectopic GMC-like cells phenotype as the number of cells negative for Dpn (red) and Elav (blue) plummeted to *wt* level (arrowheads). (E–E′) showed a control *grh^RNAi^* clone with its GMCs pointed out by the arrowheads, at 96 h ALH. (F–I″) Over-expression of *grh* in *ptc^S2^* clones substantially rescued the Mira delocalization and Dpn-expression defects in the interphase NBs at late L3 stage. More than 60% of the NB within *ptc^S2^* clones (marked by CD8:GFP in green) displayed weak cortical Mira (red) and low intensity of nuclear Dpn (blue) as compared to the surrounding *wt* NBs (F–F″), while the rest of the interphase *ptc^S2^* NBs had largely normal Mira localization and nuclear Dpn intensity (G–G″). *ptc^S2^* clones that over-expressed *grh* had a higher frequency NBs with normal Mira localization and nuclear Dpn intensity (H–H″), whereas control NB that over-expressed *grh* was indistinguishable from *wt* NB in terms of Mira localization and nuclear Dpn intensity (I–I″). Scale bar = 10 µm.

To substantiate a mechanistic link between Hh signaling, Grh expression as well as the temporally regulated cell cycle exit, we reduced the level of Grh by RNA-mediated interference in *smo^IA3^* clones. At 24 h APF, 68.5%±7.8% of *smo^IA3^* clones (*n* = 54) contained a NB that was positive for the proliferative marker Mira (Figure 7A). In contrast, expression of *grh^RNAi^* within *smo^IA3^* background significantly reverted this NB cell cycle termination defect by bringing down the frequency of clones with a proliferating NB at 24 h APF to 17.6%±9.9% (*n* = 68, *p* = 0.009), a level that was comparable to that of the control *grh^RNAi^* clones (21.6%±10.9%, *n* = 51, *p* = 0.07) (Figure 7B and 7C). Moreover, the number of GMC-like cells at 96 h ALH, which is indicative of the proliferative capacity of the NBs, was reduced to 3.4±1.2 cells per *grh^RNAi^* expressing *smo^IA3^* clone (*n* = 18, *p*<0.0001), as compared to 6.7±2.6 cells per *smo^IA3^* clone and 2.7±1.3 cells per control *grh^RNAi^* clone (Figure 7D and 7E). On the other hand, the NB proliferative defect seen in *ptc^S2^* clones was only marginally modulated by *grh* ectopic expression. Compared to *ptc^S2^* mutant NBs with a mitotic index of 21.6%±8.4%, *ptc^S2^* mutant NBs with forced *grh* expression exhibited a mitotic index of 26.1%±9.8% (*n*>45, *p* = 0.23). Hence, it is conceivable that Hh signaling might regulate NB behavior by other mechanisms in addition to Grh. Despite that, we noted that Mira delocalization defect was significantly rescued with 69.7%±18.4% of the *ptc^S2^ ;grh^o/e^* NBs showing proper Mira localization (*n* = 96, *p* = 0.008) as compared to only 37.8%±19.6% among the corresponding *ptc^S2^* NBs (*n* = 142) (Figure 7F–7I). Similarly, the percentage of *ptc^S2^* NBs with *wt*-level of *dpn*-expression intensity improved from 32.2%±15.3% (*n* = 59) to 61.5%±10.1% with simultaneous induction of *grh* expression (*n* = 65, *p* = 0.02) (Figure 7F–7I). Thus, Hh signaling regulates NB cell cycle exit, partly via *grh* expression, in conjunction with the temporal series.

### Links between the Temporal Cascade, Hh Signaling, and the Asymmetric Division Machinery

**Figure 8:**
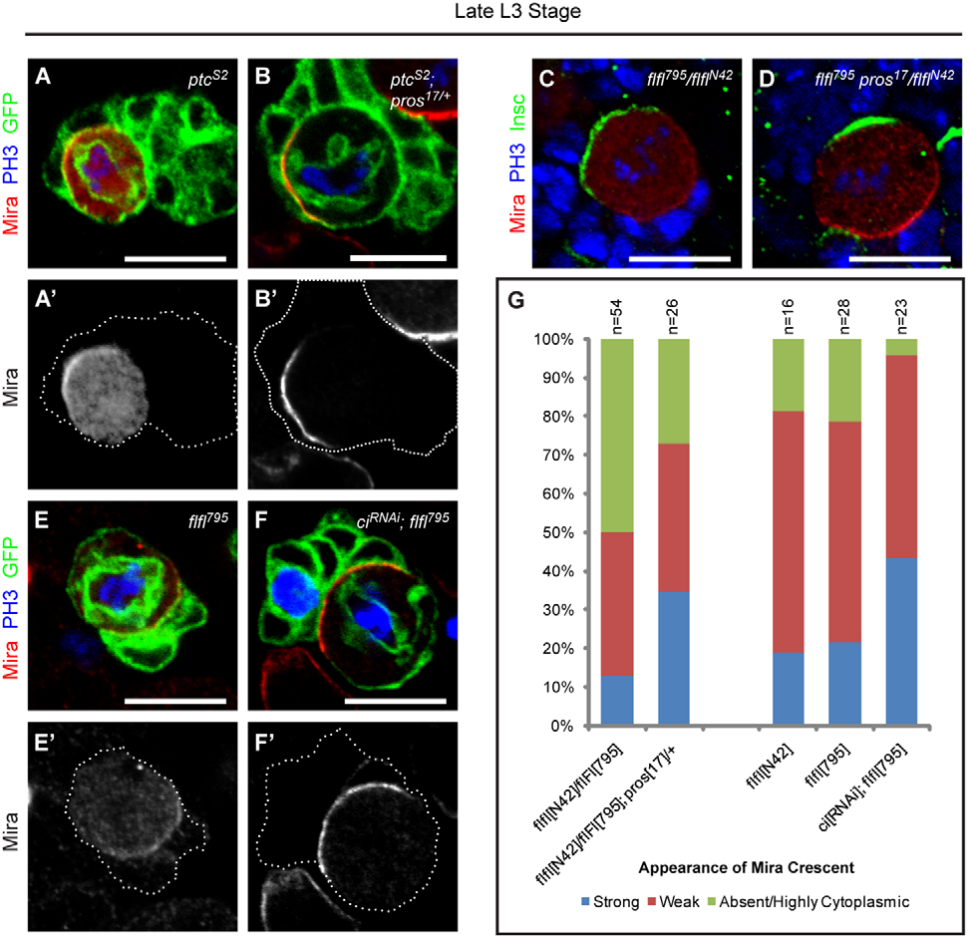
Hh signaling acts as a functional link between the temporal cascade and the asymmetric division machinery. (A–B′) The excessive cytoplasmic Mira and weak Mira crescent seen in *ptc^S2^* NB during mitosis (A–A′) can be rescued by removing one copy of *pros* (B–B′). (C–D) Similarly, the excessive cytoplasmic Mira and weak Mira crescent seen in *flfl^795^/flfl^N42^* transheterozygous NB (C) can be rescued by removing a copy of *pros* (D). (E–F′) A *flfl^795^* NB showing weak Mira crescent and cytoplasmic Mira (E–E′). Such Mira localization defects can be rescued via the introduction of *ci^RNAi^*. (G) Quantification of Mira localization phenotypes in various mutant backgrounds.

On one hand, Pros as a component of NB asymmetric division machinery, is normally tethered onto the cortex by Mira and kept out of the NB nucleus. On the other hand, a burst of interphase nuclear Pros (accompanied by cytoplasmic localization of Mira) is triggered by the temporal mechanism during the terminal division of NBs, indicative of cell cycle exit [44]. However, how the temporal mechanism can mediate nuclear Pros localization in NB is unknown. *ptc^S2^* NBs were associated with a higher level of nuclear Pros during interphase and cytoplasmic Pros during mitosis (Figure 2E, S10A–S10D). Pros functions by repressing genes required for NB self-renewal and activating genes implicated in neuronal differentiation [28]. Hence, we reasoned that the slower mitotic cycle and premature cell cycle exit in *ptc^S2^* NBs may be a result of mislocalized/nuclear Pros. Indeed, removal of one copy of functional *pros* largely suppressed the Mira mislocalization phenotype associated with *ptc* loss-of-function (Figure 8A and 8B). As many as 71.4% of the *ptc^S2^*; pros^17/+^ NBs (*n* = 21) expressed cortical Mira during interphase and strong Mira crescent during mitosis. Furthermore, the number of Dpn-negative, Elav-negative GMC-like cells was reverted to the *wt* level of 5.0±1.5 cells per clone (Figure S9A and S9B). As a control, clones with a single copy of functional *pros* had an average of 4.1±1.0 GMC-like cells (*n* = 40), showing that decreased level of *pros* itself in a *wt* background did not cause GMCs to over-proliferate (Figure S12C).

Like *ptc^S2^*, mutations in the subunits of PP4 complex had been reported to exhibit a NB under-proliferation phenotype with similar defects in the localization of Mira and Pros [32]. The majority of mitotic NBs in *flfl^N42^/flfl^795^* (the regulatory subunit of PP4) trans-heterozygotes exhibited nuclear Pros during interphase ([32] and unpublished data) and lacked a well-defined Mira crescent but instead, showed cytoplasmic accumulation of Mira during mitosis (Figure 8C and 8G). Like *ptc^S2^* NBs, removal of one copy of *pros* in *flfl^N42^/flfl^795^* trans-heterozygotes could partially restore Mira crescent to the cortex as the percentage of NBs with strong Mira crescent increased from 13.0% to 34.6% (Figure 8D and 8G). As PP4 is known to be a Smo phosphatase that dephosphorylates Smo thus dampening Hh signaling [67], abolishing the function of PP4 will invariably lead to elevated Hh signaling. Supporting the idea that excess Hh signaling may in part be the cause for Mira/Pros delocalization in *flfl* mutants, we knocked down *ci* by RNAi in the *flfl^795^* background and observed that the number of NBs with strong Mira crescent increased from 21.4% to 43.5%, while NBs expressing *ci^RNAi^* did not exhibit any noticeable phenotype in that respect (Figure 8E, 8G, and unpublished data). Thus, our data suggest that PP4 regulates NB divisions, in part, by dephosphorylating Smo and modulating Hh signaling to keep NBs in a proliferative state. Supporting this, compromising PP4 function suppressed the formation of expanded GMC-like pools and promoted cell cycle exit in a *cas* mutant background (Figure 4I–4K). Together, our results suggest that Hh signaling plays a role in NB asymmetric division via the regulation of Mira/Pros localization; it also acts both downstream (*cas*) and upstream (*grh*) of components of the temporal series to control NB cell cycle exit. Thus Hh signaling appears to be a key player in coordinating the asymmetric division machinery with the temporal cascade to schedule the termination of postembryonic neurogenesis in line with developmental timing.

## Discussion

**Figure 9:**
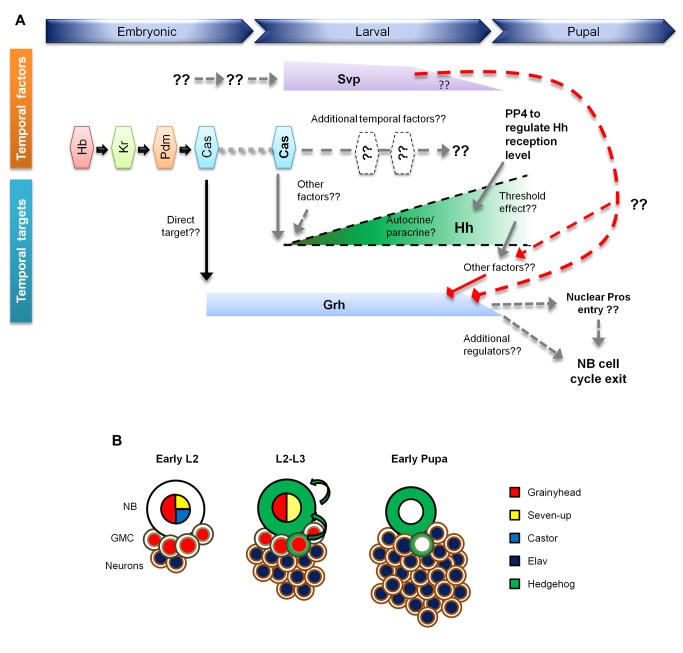
The model. (A) An early pulse of Cas at early larval stage primes the expression of *hh* in both NB and GMCs. The Hh ligand (unpublished data) acts in an autocrine and/or paracrine fashion to activate Hh signalling transduction in the NB. Among the outcomes of Hh signaling pathway activation in the context of postembryonic NB, *grh* expression is down-regulated and Pros moves into the nucleus, eventually leading to NB cell cycle exit at early pupal stage. The strength of Hh signaling activation is likely to be regulated PP4, which dampen Hh pathway activity by dephosphorylating Smo. *svp* probably constitutes a parallel pathway, which may or may not converge with *cas*→*hh* pathway at a point prior to the eventual NB cell cycle exit. Dotted lines and double question marks denote uncertainties while solid lines represent availability of experimental evidences. Triangular and diamond-shaped arrowheads imply positive and negative regulations respectively. This illustration is not drawn to scale. (B) Type I NB lineage trees at three different developmental stages, during which the expression of Grh (red), Cas (blue), Svp (yellow), Hh (green), and Elav (dark blue) are shown. The NB, GMC, and neuron are represented by circles with black, tan, and brown outlines, respectively. The green arrows show the autocrine/paracrine mode of Hh signaling during lineage progression.

Here, we show that Hh signaling functions during later postembryonic development and acts together with the NB temporal transcription factor cascade to regulate NB cell cycle exit (Figure 9). We further demonstrate that *hh* is a downstream target of Cas, a member of temporal series that determines the time at which NBs terminate proliferation via down-regulation of Grh. While increased Hh signaling results in increased cell cycle length and premature NB cell cycle exit, loss of Hh signaling decreases NB cell cycle length and also prolongs the duration of NB proliferation.

### Hh Acts at Short Range in the Larval Brain

Hh family proteins can act as short- or long-range morphogens covering distances as few as ten cell diameters (20 µm), or as far as a field containing many more cell diameters (200 µm) [68],[69]. In the postembryonic brain, *hh* is expressed predominantly in the NBs and the newborn GMCs, whereas the expression of its target gene reporter, *ptc-lacZ* is observed in a narrow area covering the adjacent NB and the sibling GMCs, indicating a limited response to and suggesting a limited spread of Hh ligand. In addition, Hh protein is always found to be enriched and clustering around the NBs in a punctuated form rather than forming a gradient. These data, together with the lineage autonomous phenotype of *hh* mutant NB clones, strongly suggest that Hh acts locally at short range in the larval brain. This is consistent with the structural arrangement of the larval brain, where each NB lineage comprising of the NB itself, GMCs, and neurons, is encapsulated by a meshwork of glial processes that form a three-dimensional scaffold that potentially acts as a stem cell niche [70],[71]. Such a spatial arrangement may serve as a barrier to restrict spread of the ligand and confine signaling events within a particular lineage so that an individual NB lineage can development with considerable independence from its neighbouring lineages. Indeed, a NB clone derived from a *hh* null allele exhibits the GMC pool expansion phenotype even though GMCs from its neighbouring lineages are competent in producing Hh ligand.

While it is tempting to assume that Hh can also act on the GMCs in an autocrine mode of action judging from the presence of *ptc-lacZ* expression, we did not observe any noticeable GMC fate transformation or change in their proliferative capability in *ptc^S2^* and *smo^IA3^* clones. The higher mitotic rate in *hh* loss-of-function NBs could largely explain the amplification of the GMC pool and enlarged clone-size; however, we are unable to rule out a possible delay in GMC differentiation. The proposition that Hh ligand, which is produced by the NB and daughter GMCs, feeds back on the NB to control its own proliferative capacity and the timing of cell cycle exit is interesting but not totally unfamiliar. Similar feedback signalling mechanism has been demonstrated in the mouse brain in which post-mitotic neurons signal back to the progenitor to control cell fate decisions, as well as the number of neurons and glia produced during corticogenesis [72].

### Temporal Regulations of Hh Signalling

Hh signal reception is detectable in NBs as early as in L2 and persists throughout larval life and in early pupae when NBs undergo Pros-dependent cell cycle exit. This delay of approximately 96 h between the start of Hh reception and the ultimate outcome of cell cycle exit may be due to a requirement for cumulative exposure of NBs to increasing local concentrations of Hh. Such a graded response will enable the *wt* postembryonic NBs to progress from high to low proliferative stages before ceasing division, in line with the development of the larva. Evidence supporting this notion includes gradual accumulation of Hh on the NBs, lengthening of NB cell cycle time, as well as the necessity of high levels of Hh signaling to trigger cell cycle exit (unpublished data). It is worthwhile to note that even at pre-pupal stage during which most NBs are starting to undergo cell cycle exit, fewer than 20% of them are associated with Hh puncta at any point of time. One likely explanation is that not all the NB lineages within the larval central brain respond synchronously to Hh-mediated temporal transition. However, unlike the embryonic central nervous system in which *hh* expression is localized to rows 6–7 of the neuroectoderm [73], we find it difficult to pinpoint a specific expression pattern in the postembryonic central brain due to the disorganized array of NB lineages. It is equally possible that different NBs exit cell cycle progression at different time points. This is also consistent with the structural organization of individual NB into different “trophospongium” or stem cell niches. Nevertheless, we cannot rule out the possibility that Hh signal activation primes another yet-to-be-identified developmentally regulated signal/event to schedule NB cell cycle exit.

Interestingly, a recently proposed “cell cycle length hypothesis” postulates that cell cycle length, particularly the length of G1 phase in neural stem cells acts as a switch to trigger the transition from proliferative to neurogenesis mode [62]. In fact, experiments have shown that manipulation of cdk4/cyclinD1 expression and cdk2/cyclinE activity that result in the lengthening of G1 is sufficient to induce precocious neurogenesis; while inhibition of physiological lengthening of G1 delays neurogenesis and promotes expansion of intermediate progenitors [74],[75]. Our results show that *Drosophila* postembryonic NBs in the central brain exhibit a comparable trend of cell cycle lengthening from young to old larval stages. Interestingly, NBs with excess Hh signaling have an extended cell cycle time, consistent with the idea that there is a forward shift of the “perceived” age, leading to premature cell cycle exit. In contrast, Hh loss-of-function NBs have a shorter cell cycle time compared to their *wt* counterparts of the same actual age; hence, they have a younger “perceived” age and are able to maintain their proliferative phase over a longer period of time. Consistent with this, we showed that persistent NB proliferation in *smo^IA3^* clones as well as the early termination of *ptc^S2^* NBs proliferation, are always associated with the presence and absence of CycE expression, respectively. However, loss of Hh signaling in NBs merely extends their proliferative phase but is not sufficient to ensure perpetual proliferation as we failed to observe any mitotic NB in the adult brain. We also note that a previous report suggested that the cell cycle time of the larval NBs reduced during their growth and reached a peak at late third instar with a minimum cell cycle time of 55 min. However, this study was conducted on thoracic NBs from the neuromeres T1 to T3, which have a very distinctive proliferative profile to the central brain NBs assayed in the current study [6]. Indeed, it was shown in their study that abdominal NBs exhibited significantly different cell cycle times compared to their thoracic counterparts.

In *Drosophila*, the precise timing of NB cell cycle exit is governed by a highly regulated process that involves sequential expression of a series of transcription factors: Hb→Kr→Pdm1→Cas, known as the temporal series [36],[37],[76]. It is known that the temporal series probably utilizes Grh in the postembryonic NBs to regulate Pros localization or apoptotic gene activity, thus determining the time at which proliferation ends. In addition, the temporal series also regulate postembryonic Chinmo→Br-C neuronal switch, which specifies the size and the identity of the neurons [44],[77]. Our data show that Hh signaling does not regulate early to late neuronal transition as Chinmo and Br-C expression timings appear unaffected in both *ptc* and *smo* mutant clones (unpublished data). In contrast, excess Hh signaling leads to a variety of features associated with NB cell cycle exit: (1) premature down-regulation of Grh, (2) nuclear localization of Pros (in NBs), and (3) reduction of NB size. Taken together with the extended proliferative duration of Hh loss-of-function NBs, it is apparent that Hh signaling is a potent effector of the temporal series and functions late to promote NB cell cycle exit.

The results from our current genetic interaction assays with Hh pathway components and *grh* reaffirmed the conclusions from previous studies that Grh is necessary to maintain the mitotic activity of the postembryonic NBs [42],[44]. The loss of Hh signaling keeps the central brain type I NBs in their proliferative state and this is largely contributed by persistent *grh* expression past their normal developmental timing at around 24 h APF. Even though Grh is necessary to extend the proliferative phase of these NBs, it is not sufficient to rescue all aspects of the premature cell cycle exit phenotype seen in *ptc* mutant NBs. Hence, down-regulation of *grh* by over-activating Hh signaling is not solely responsible for NB proliferative defects, and this implies that Hh signaling may terminate NB cell cycle via other mechanisms in addition to Grh.

The expression of *hh* appears to be dependent on the pulse of Cas expression at the transition between L1 and L2, as induction of *cas* mutant clones after that stage does not significantly affect *hh* expression. Moreover, ChIP assays suggest that Cas binds the *hh* genomic region, thereby placing Hh as a direct downstream target of the temporal series. However, it is intriguing to speculate on how the early pulse of Cas can mediate *hh* expression, which only comes on later during larval development. One possible explanation involves a relay mechanism in which that pulse of Cas activates an (or a cascade of) unknown components, which persist and eventually turns on the later *hh* expression. Yet, in such a model, Cas need not interact directly with the *hh* locus as our ChIP assay clearly suggests. Moreover, there are at least two pulses of *hh* expression during larval brain development, and the earlier, shorter pulse that is required for the activation of quiescent NBs appear to be independent from Cas regulation as Cas is only switched on in the larval NBs upon reactivation [70]. Most importantly, our data show that mis-expression of *cas* abolishes, rather than triggers ectopic *hh* expression. Thus, our findings do not favour the continuous expression of a *hh* activator downstream of Cas. Alternatively, Cas may be involve in the epigenetic modifications of the *hh* locus such that it is primed for expression at a much later stage. This may also explain why saturating the system with Cas for prolonged period of time via mis-expression can negatively affect subsequent *hh* expression because of to its potential aberrant association with the chromatin. Although such a function has not been reported for Cas, previous studies have postulated that components of the temporal series, such as Hb (or mammalian homolog Ikaros) and Svp (or mammalian homolog COUP-TFI/II), play a role in modulating chromatin structure, hence modifying the competency of downstream gene expression subsequently [78]–[80].

The relationship between *svp* and Hh signaling within the postembryonic temporal series cascade is interesting yet unexpected. *svp* was thought to be a downstream component of *cas* on the basis of studies in postembryonic NBs in the thoracic segment of the ventral nerve cord [44]. This is supported by the observations that the pulse Svp occurs at 40–60 h ALH following the pulse of Cas at 30–50 h ALH. Moreover, both *svp* and *cas* mutant clones affect Chinmo/Br-C neuronal target transition, apart from causing NBs' failure to exit the cell cycle at early pupal stage. However, examinations of Svp and Cas expression patterns in the central brain region in this study reveal that the Cas expression window overlaps with the peak of the Svp expression window, even though the latter has a much wider expression window in which low expression levels can still be detected in the NBs at 96 h ALH. Moreover, our data show that abolishment of *cas* function starting from the embryonic stage does not reduce Svp expression in the NBs at 24 h ALH. Hence, previous interpretation that *svp* functions downstream of *cas* in the thoracic postembryonic NBs may not be easily extrapolated to NBs in other brain regions. On the basis of our results, it is tempting to postulate that Cas and Svp constitute two parallel pathways within the temporal series and Hh signaling is regulated by Cas but not Svp. Nevertheless, such a hypothesis warrants more in depth studies.

### Hh Signaling Provides a Link between NB Asymmetry and the Temporal Series

The precise generation of diverse cell types with distinct function from a single progenitor is important for the formation of a functional nervous system during animal development. It has been shown that, in *Drosophila*, the developmental timing mechanism (the temporal series) is tightly coupled with the asymmetric machinery [44]. However, the underlying mechanism of this coordination remains elusive. Our data suggest that on the one hand, Hh signaling is under the control of the temporal series (*hh* expression is directly regulated by Cas), while on the other hand, Hh signaling participates in asymmetric segregation of Mira/Pros during NB division. Introduction of ectopic/premature Hh signaling (in *ptc* mutant clones) during developmental stages in which NBs are proliferating results in cytoplasmic localization of Mira/Pros during mitosis, reduction of NB size, and slow-down of NB cell cycle progression, reminiscent of the final division of NBs in early pupa just before cessation of proliferation. Consequently, these NBs exit the cell cycle prematurely. We speculate that Pros may be a direct or indirect target of Hh signaling as elevated pathway activity invariantly leads to increased *pros* expression in the NBs. Furthermore, reducing the level of Pros protein by removing one copy of function *pros* is able to rescue the Mira delocalization phenotype seen in *ptc* mutant NBs. Thus, it is plausible that Hh signaling impinges on the asymmetric division apparatus, likely through Pros, to diminish NB fate gradually (as seen with the absence of Dpn and Mira delocalization) prior to the final cell cycle exit. Despite our results indicating a tight correlation between nuclear entry of Pros into the NBs and the eventual cell cycle exit of these NBs during pupal stage, we would like to caution the readers that Pros may not be the direct causative agent in controlling NB cell cycle exit. Therefore the actual role of Pros in this process is purely speculative as far as this study is concerned.

On the other hand, loss of Hh signaling (e.g., in *Smo* mutant clones) maintains NBs in their “younger” proliferating stage far beyond the time when they normally exit the cell cycle. Thus, Hh signaling couples the developmental timing mechanism (the temporal series) with the NB intrinsic asymmetric machinery for the generation of a functional nervous system.

### The Anti-proliferative Role of Hh Signaling

In vertebrates, constitutive activation of the Sonic hedgehog (SHH, a homologue of *Drosophila* Hh), signaling pathway through inactivation mutations in PTCH1, activating mutations in SMO, as well as other mutations involving SHH, IHH, GLI1, GLI2, GLI3, and SUFU, has been implicated in a vast array of malignancies [81],[82]. The proven association of Hh signaling pathway with tumourigenesis and tumour cell growth fuel the view that Hh constitutes a mitogenic signal that promotes pro-proliferative responses of the target cells. Moreover, Hh acts as a stem cell factor in somatic stem cells in the *Drosophila* ovary, human hematopoietic stem cells, and mouse embryonic stem cells, possibly by exerting its effects on the cell cycle machinery [83]–[86].

Our report here provides an opposing facet of Hh signaling where it is required for timely NB cell cycle exit in the postembryonic pupal brain. This may sound astonishing, but the essential roles of Hh signaling as a negative regulator of the cell cycle has been eclipsed by the common bias that it stimulates proliferation, given the many examples of malignancies with the Hh pathway dysregulation. Indeed, studies have indicated that cell cycle exit and differentiation of a number of cell types, such as absorptive colonocytes of the mammalian gut, zebrafish, and *Drosophila* retina, require Hh activities [87]. A more recent article also showed that SHH signaling pathway is highly activated in human embryonic stem cell (hESC) and such activity is crucial for hESC differentiation as embryoid bodies [88]. The opposing functions of Hh signaling pathway in different cell types reveal that the ultimate effect of this pathway is likely to be tissue specific, depending on its interaction with other regulatory pathways. Our data indicate that in *Drosophila* postembryonic NBs of the brain this does indeed appear to be the case, because in this system, Hh signaling pathway interacts with NB-specific temporal series and likely the asymmetric cell division machinery to promote *pros* nuclear localization to trigger cell cycle exit.

## Methods

### *Drosophila* Strains

All fly stocks and crosses were maintained at 25°C. Stocks used were *FRT40A*, *FRT42D*, *FRT82B*, *ptc^S2^*, *ptc^13^* (P. Ingham), *smo^IA3^*, *ci^94^* (K. Basler), *cas^24^* (A. Gould), *pros^17^*, *flfl^N42^*, *flfl^795^*, G147, *svp^1^*, *grh^RNAi^* (Bloomington, 33678/GD), *ptc^RNAi^* (Bloomington, JF03223), *PP4-19C^RNAi^* (VDRC, 25317/GD), *ci^RNAi^* (VDRC, 51479/GD), *elav-GAL4*, *UAS-CD8::GFP*, *UAS-ci^Nc5m5m^* (D. Kalderon), *ptc-LacZ* (J. Hooper), and *UAS-grh* (S. Thor), *UAS-histone2AvRFP* (M. Gonzalez-Gaitan), *UAS-svp1.12* (Y. Hiromi), *UAS-stg.N4*, *UAS-cycE.L*, *UAS-smo^RA1234^* (J. Jiang), *UAS-histone::RFP* (J. Bellaiche), *UAS-pon::GFP* (B. Lu), *Ay-GAL4* (*act* flip-out). All stocks were obtained from Bloomington Stock Center unless otherwise stated.

### Clonal Analysis

Embryos were collected over a period of 6 h, heat-shocked in 37°C water bath at 24 h and 48 h ALH for all experiments unless otherwise specified, and larvae and pupae of desired genotype (see below) were dissected at specific time points and processed for immunochemistry analysis. Under our culture conditions, *Drosophila* larvae underwent approximately 108 h of postembryonic development. After hatching from the embryo, the 1st-instar larva (L1) stage lasted for 24 h before molting into 2nd-instar larva (L2). The L2 to 3rd-instar larva (L3) transition occurred at approximately 48–60 h after hatching, and finally L3 larva pupate at 96–108 h after hatching. MARCM clones were generated according to the technique reported previously [55].

### Immunohistochemistry and Imaging

Brains were fixed for 15 min in 3.7% formaldehyde in PBS with 0.1% Triton-X. The following antibodies were used: mouse anti-Mira (F. Matsuzaki), 1/50; rabbit anti-Mira (generated in our lab), 1/1,000; chicken anti-green fluorescent protein (GFP) (Abcam), 1/2,000; guinea-pig anti-Dpn (J. Skeath), 1/500; mouse anti-Pros (DSHB), 1/10; rat anti-Elav (DSHB), 1/5; mouse anti-BrdU (Sigma), 1/50; rat anti-Hh (I. Guerrero), 1/20; rabbit anti-Grh (B. Bello), 1/200; rabbit anti-Pon (Y.N. Jan), 1/500; rabbit and mouse anti-phosphohistone H3 (Abcam); rabbit anti-aPKCζ C20 (Santa Cruz Biotechnologies), 1/1,000; rabbit anti-Pins, 1/1,000; rabbit anti-Insc, 1/1,000; guinea-pig anti-Numb (J. Skeath), 1/1,000. Secondary antibodies were conjugated to either Alexa Fluor 488, Alexa Fluor 555, or Alexa Fluor 633 (Molecular Probes), and used at 1/500, 1/1,000, and 1/250, respectively. DNA stain was To-PRO-3 (Molecular Probes), 1/5,000 and samples were mounted in Vectashield (Vector Laboratories). Images were obtained using Zeiss LSM 510 upright microscope and processed in Adobe Photoshop CS3 and Adobe Illustrator CS3.

### Live Imaging

Brains were dissected from larvae at 48 h, 72 h, and 96 h ALH, and were prepared for live imaging using the clot method as describe previously [89],[90]. Image acquisition was performed at 25°C on Leica SP5 inverted microscope. Multiple z-sections were recorded with step-size of 2–4 µm. Each z-stacks was recorded every 5 min over a period of 6–8 h in order to capture at least one complete cell cycle (only the first cell cycle will be considered for calculation of cell cycle length). Images obtained were processed using Adobe Photoshop CS3 and ImageJ. Refer to Text S1 for genotypes of the larvae used.

### BrdU Incorporation

Larvae at the age of 82–88 h ALH were picked up from the fly food and starved for 1 h on a clean Petri dish. They were then fed with yeast paste infused with 0.1 mM BrdU (Roche) for 4 h before being dissected and analyzed.

### In Situ Hybridization

*hh* genomic region was amplified with Expand High Fidelity PCR system (Roche) to yield a ∼1.9-kb intronic and a ∼0.7-kb exonic template using the following primer-pairs:

*Intronic*: 5′-GTGGATTTGGATCTGGCTATC-3′ and 5′-CAATTAGCCGCGATACAGCAC-3′

*Exonic*: 5′-ATTCGTCGATCAGTTCCCACGTGC-3′ and 5′-GATGGAATCCTGGAAGAGCGATCC-3′

Digoxigenin-labeled probes were generated using DIG RNA Labeling kit according to the manufacturer's instructions (Roche). Larval brains of specific genotype were rinsed thoroughly with PBS and dissected at specific age. They were fixed for 20 min in 3.7% formaldehyde in PBS supplemented with 0.1% Tween-20. In situ hybridization was performed as previously described [91],[92].

### Plasmid and Cloning

Full length cDNA of *cas* was amplified from DGC gold collection (LD36057, BDGP) using Expand High Fidelity PCR system (Roche) with the primers: 5′-ATGTCCAACCAAATGGAGTTTA-3′ and 5′-CTACTCCTTAAACTCTGGCTTAAAGCT-3′. The resultant PCR product was TOPO-cloned into pENTR vector (Invitrogen) and switched into pAFW vector (Drosophila Gateway Vector collection) using the pre-existing protocol (T. Murphy). The flag-tagged Cas construct was fully sequenced.

### S2 Cells Transfection

2×10^6^ S2 cells were seeded onto a 75-ml culture flask at 25°C a day prior to transfection. 2.5 µg of Flag epitope tagged Cas construct was transfected into these cells using Qiagen Effectene transfection reagent. DNA to effectene ratio was maintained at 1∶20. 24 h post-transfected cells were used for ChIP.

### Chromatin Immunoprecipitation

ChIP was performed according to the manufacturer's protocol for EZ-Magna ChIP G (Milipore).

### Quantitative PCR

qPCR was performed using KAPA SYBR FAST qPCR kit (KAPA Biosystems) according to the standard protocol on 7900HT Fast Real-Time PCR system (Applied Biosystems). The sequences of the primer-pairs used are listed in Text S1.

## Supporting Information

Figure S1 **Excess Hedgehog signaling only affected the localization of the Mira/Pros complex.** (A–D′) *wt* NBs formed apical crescents of aPKC (A–A′, red), Pins (B–B′, red), and Insc (C–C′, red), as well as basal crescents of Mira (C, C″, blue), and Numb (D–D′, red) during mitosis. (E–H′) *ptc^S2^* NBs localized aPKC (E–E′, red), Pins (F–F′, red), Insc (G–G′, red), and Numb (H–H′, red) correctly during mitosis, but often delocalized Mira into the cytoplasm (G, G″, blue). Note that Insc crescent was slightly weaker in *ptc^S2^* NBs compared to *wt* NBs. (I–L′) *smo^IA3^* NBs formed *wt* crescents of aPKC (I–I′, red), Pins (J–J′, red), Insc (K–K′, red), Mira (K, K″, blue), and Numb (L–L′, red) during mitosis. (M–M′) A mitotic *ptc^13^* NB (marked by GFP) located next to a *wt* NB. Note that Mira (red) was largely delocalized into the cytoplasm in *ptc^13^* NB, as opposed to the strong Mira crescent seen on the cortex of the *wt* NB. Scale bar = 10 µm.
(TIF)

Figure S2 **Mis-expression of** ***pros*** **abolished** ***dpn*** **expression and mislocalized Mira in the NBs.** (A–A′) *act*-Gal4 driven *pros* expression in flip-out clones (marked by CD8:GFP in green) resulted in down-regulation of nuclear Dpn (red) in the NB, while neighbouring *wt* NBs exhibited strong nuclear Dpn (arrows) after 12 h of clonal induction. (B–B′) In the same clonal background, cortical Mira (red) was absent from an interphase NBs (as judged by the lacked of PH3 in blue), while two neighbouring NBs, in interphase (arrow) and metaphase (arrowhead), showed normal cortical enrichment of Mira. Scale bar = 10 µm.
(TIF)

Figure S3 **All cells in** ***smo^IA3^*** **mutant clones expressed neuronal marker in adult brain.** (A–A″) All the cells within *smo^IA3^* clone (marked by CD8:GFP in green) in 1-d-old adult brain were Elav positive. (B–D′) MARCM clones for *ptc^S2^; smo^3/+^* (marked by CD8:GFP in green) in late third instar larval brain. (B–B′) An example of a clone that contains four GMC-like cells (arrows) that were Dpn- (red) and Elav- (blue) negative. The mitotic NBs (as shown by the expression of PH3, blue) showed distinct Mira (red, C–C′) and Pros (red, D–D′) crescents. Scale bar = 10 µm.
(TIF)

Figure S4 **Hh ligand acted in a lineage restricted manner.** (A) *wt* NB clone (marked by CD8:GFP, green) with four undifferentiated GMC-like cells, which were both Dpn- (red) and Elav- (blue) negative (arrows) as compared to (B–B′) *hh^AC^* clone, which showed six undifferentiated GMC-like cells (arrows; arrowhead marks one GMC that was partially hidden from view). (C–C″) Three consecutive *z*-sections (6 µm apart from each other) of a single *hh^AC^* clone. Scale bar = 10 µm.
(TIF)

Figure S5 **High levels of Hh signaling led to nuclear Pros localization in NBs.** (A–B′) *act-Gal4* flip-out driver induced clones (marked by CD8:GFP, green) that ectopically expressed *smo^RA1234^* (A–A′) and *ci^5M^* (B–B′). The NBs (Dpn positive, blue) within the clones showed weak nuclear localization of Pros (red, arrowheads), while the neighbouring *wt* NB was devoid of nuclear Pros (arrow). Scale bar = 10 µm.
(TIF)

Figure S6 **Hh signaling was perceived by the NBs.** (A) The percentage of the NB with bound Hh was determined by calculating the number of NBs with bound Hh over the total number of NBs in the central brains of *wt* larvae at different age windows. Error bars corresponds to standard error of the mean (SEM). (B) Accumulation of Hh protein on/within the *wt* NBs (outlined by GFP, green) at 96 h ALH was visualized with anti-Hh antibody (red). (C) A *wt* third instar larval brain lobe was immunostained to show the expression of Dpn (red), Elav (blue), and the Hh reception reporter, *ptc*-lacZ (green). Inset showed two separate NB clones in which β-Gal expression was detected in the NB and, to a lesser extent, GMCs. Scale bar = 10 µm.
(TIF)

Figure S7 **Proliferative status of the NB correlated with CycE expression.** (A–C′) MARCM clones (marked by CD8:GFP, green) for *wt* (A–A′) and *smo^IA3^* (C–C′) in late third instar larval brains contained a single Dpn- (blue) positive NB that co-expressed CycE (red). (B–B′) The expression of CycE was largely abolished in *ptc^S2^* NB (arrowhead) as compared to the surrounding wt NBs outside the clone (arrows). (D–E′) At 24 h APF, *smo^IA3^* (D–D′) and *cas^24^* (E–E′) clones (marked by CD8:GFP, green) continued to express Dpn (blue) and CycE (red) when most of the surrounding wt NBs had already down-regulated both Dpn and CycE. Scale bar = 10 µm.
(TIF)

Figure S8 **Mis-expression of** ***cas*** **crippled** ***hh*** **expression at 96 h ALH.** (A–B′) In situ hybridization of hh mRNA (red) showed that mis-expression of *cas* in *act*-Gal4 flip-out clones (marked by CD8:GFP, green) affected *hh* expression when induced at both embryonic stage (A–A′) and late L2 stage (B–B′). Pon (blue) showed the outline of the newly born GMCs, which typically expressed *hh* mRNA. Note that the GMCs within the clones that mis-expressed *cas* (arrowheads) were devoid of hh transcript, while most of the surrounding *wt* GMCs (arrows) expressed *hh* normally. (C–C′) A clone that mis-expressed *cas* (CD8:GFP, green) continued to harbor a Mira-positive NB (blue) at 24 h APF. Scale bar = 10 µm.
(TIF)

Figure S9 **Hh signaling and Svp were unlikely to function in a linear pathway.** (A–C′) NB clones at 40 h ALH for different genotype: *wt* (A–A′), *ptc^S2^* (B–B′), and *smo^IA3^* (C–C′) were marked by CD8:GFP in green. The NBs (labeled with Dpn, blue) within the clones (arrowheads) expressed Svp (red) in a manner that was indistinguishable from the neighbouring *wt* NBs (arrows). (D–E′) Both *svp^1^* mutant (D–D′) and *act-Gal4* flip-out driven *svp* mis-expression (E–E′) clones in the central brain (labeled by CD8:GFP, green) contained GMCs that expressed *hh* transcript (red) at 96 h ALH. (F–I′) The expression patterns of Svp in the brain lobe at various time points ALH. Svp was found to be expressed strongly in the NBs (co-labeled with Dpn, green) at 24 h (F–F′, arrowheads), and became progressively weaker as time passed: 40 h (G–G′), 60 h (H–H′), 92 h (I–I′). Svp was also found to be expressed in the neurons and glia that were non-Dpn positive. (J–J′) Embryonic clone of *cas^24^* induced at 12–16 h AEL (marked by CD8:GFP, green) expressed Svp (red) in the NB, which was also expressing Dpn (blue). (K) A box-plot that showed the expression level of Svp in the NBs at different time points, normalized to the mean of the highest Svp expression in non-NB cells. The five number summaries were minimum, lower quartile, median, upper quartile, and maximum. Scale bar = 10 µm.
(TIF)

Figure S10 **Mira and Pros failed to localize properly throughout the entire mitotic phase in** ***ptc^S2^*** **NBs.** (A–D″) *ptc^S2^* NBs (marked by CD8:GFP, green) at different mitotic stages were examined for the localization of Mira (blue) and Pros (red). Interphase NBs (A–A″) were often devoid of cortical Mira and showed abnormal nuclear accumulation of Pros. Those NBs that managed to enter metaphase (B–B″) frequently showed weak Mira/Pros crescent, along with their cytoplasmic displacement that persisted through anaphase (C–C″) and telophase (D–D″), even though size asymmetry appeared to be unaffected. (E) Interphase *ptc^S2^* NBs contained higher level of nuclear Pros, as measured by the intensity relative to neighbouring *wt* NBs. Error bars corresponds to standard deviation (SD). Scale bar = 10 µm.
(TIF)

Figure S11 **Hh signaling induced cell cycle exit in the NBs via down-regulation of Grh.** (A–B) An example of NB size (diameter) in wt (A) and *ptc^S2^* (B) clones at 96 h ALH. (C) Quantitation of NB diameter in wt, *ptc^S2^*, *ptc^13^*, and *smo^IA3^* clones at 96 h ALH. Error bars represent standard deviation (SD) while statistical significance was determined using Student's *t* test. (D–D′) *smo^IA3^* clones in the pupal brain (36 h APF) continue to express Grh (red) in NB and GMCs even though all other neighbouring wt cells had down-regulated the Grh expression. (E–E′″) *ci^94^* clones, as marked by the absence of GFP (green, panel E′) at 24 h APF frequently contained NB that continued to express Dpn (blue) and Grh (red), as well as some GMCs which were Grh-positive as well. Scale bar = 10 µm.
(TIF)

Figure S12 **The phenotype of** ***ptc^S2^*** **clones can be suppressed by removing one copy of** ***pros*** **.** (A–A″) In *ptc^S2^* clone (marked by GFP), all the cells other than the NB were expressing Elav (blue). (B–B″) The number of Dpn-negative, Elav-negative GMCs (arrows) was reverted to wt levels in homozygous *ptc^S2^* clone when one copy of *pros* is removed. (C–C″) Removal of one copy of *pros* did not cause the expansion of GMC-like cells (arrow) by itself. Scale bar = 10 µm.
(TIF)

Text S1 **Supplemental material and methods.**
(DOC)

Video S1 **Live imaging of a dividing wt NB at 72 h ALH.** Histone-RFP in red, Tub-GFP (G147) and Pon-GFP in green.
(AVI)

Video S2 **Live imaging of a dividing** ***smo^IA3^*** **mutant NB at 72 h ALH.** Histone-RFP in red, and CD8-GFP in green.
(AVI)

Video S3 **Live imaging of a dividing** ***ptc^S2^*** **mutant NB at 72 h ALH.** Histone-RFP in red, and CD8-GFP in green.
(AVI)

## Abbreviations

ALH: after larval hatching
APF: after puparium formation
aPKC: atypical protein kinase C
BrdU: 5-bromo-2′-deoxyuridine
Cas: Castor
ChIP: chromatin immunoprecipitation
Ci: Cubitus Interruptus
CycE: cyclin E
Dpn: Deadpan
GFP: green fluorescent protein
Grh: Grainyhead
GMC: ganglion mother cell
Hb: Hunchback
Hh: Hedgehog
Insc: Inscuteable
MARCM: mosaic analysis of a repressible marker
Mira: Miranda
NB: neuroblast
Pdm: POU homeodomain protein
PH3: phospho-histone 3
Pins: Partner of Inscutable
Pon: Partner of Numb
PP4: protein phosphatase 4
Pros: Prospero
Ptc: Patched
RFP: red fluorescent protein
Smo: Smoothened
Svp: Seven-up
wt: wild type
