# Supplementary figures and images for: Hedgehog Signaling Acts with the Temporal Cascade to Promote Neuroblast Cell Cycle Exit
Source: PLoS Biol. 2013 Feb 26;11(2):e1001494. doi: 10.1371/journal.pbio.1001494 (PMC3582610; doi:10.1371/journal.pbio.1001494)

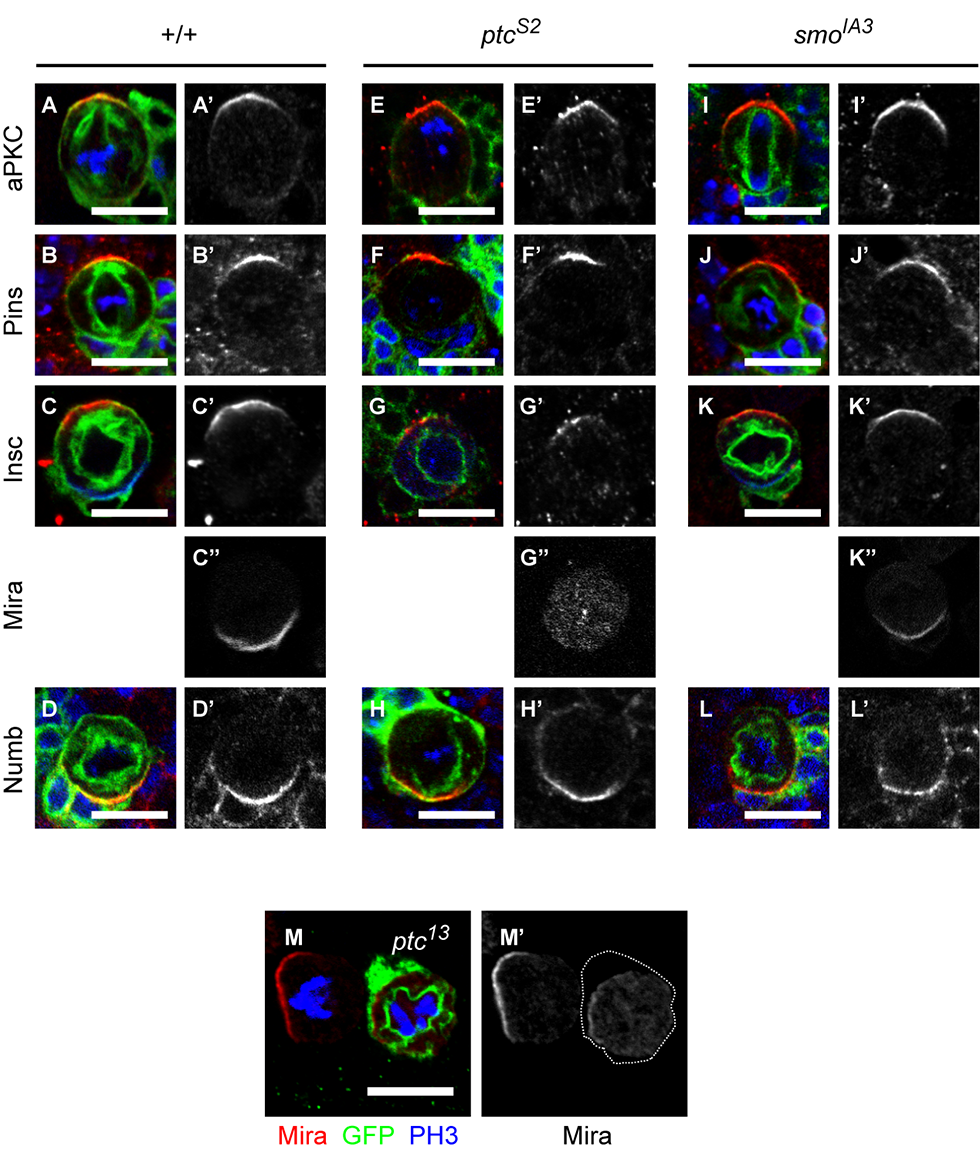

Supplement: Figure S1 — Excess Hedgehog signaling only affected the localization of the Mira/Pros complex. (A–D′) wt NBs formed apical crescents of aPKC (A–A′, red), Pins (B–B′, red), and Insc (C–C′, red), as well as basal crescents of Mira (C, C″, blue), and Numb (D–D′, red) during mitosis. (E–H′) ptcS2 NBs localized aPKC (E–E′, red), Pins (F–F′, red), Insc (G–G′, red), and Numb (H–H′, red) correctly during mitosis, but often delocalized Mira into the cytoplasm (G, G″, blue). Note that Insc crescent was slightly weaker in ptcS2 NBs compared to wt NBs. (I–L′) smoIA3 NBs formed wt crescents of aPKC (I–I′, red), Pins (J–J′, red), Insc (K–K′, red), Mira (K, K″, blue), and Numb (L–L′, red) during mitosis. (M–M′) A mitotic ptc13 NB (marked by GFP) located next to a wt NB. Note that Mira (red) was largely delocalized into the cytoplasm in ptc13 NB, as opposed to the strong Mira crescent seen on the cortex of the wt NB. Scale bar = 10 µm. (TIF) [file pbio.1001494.s001.tif]

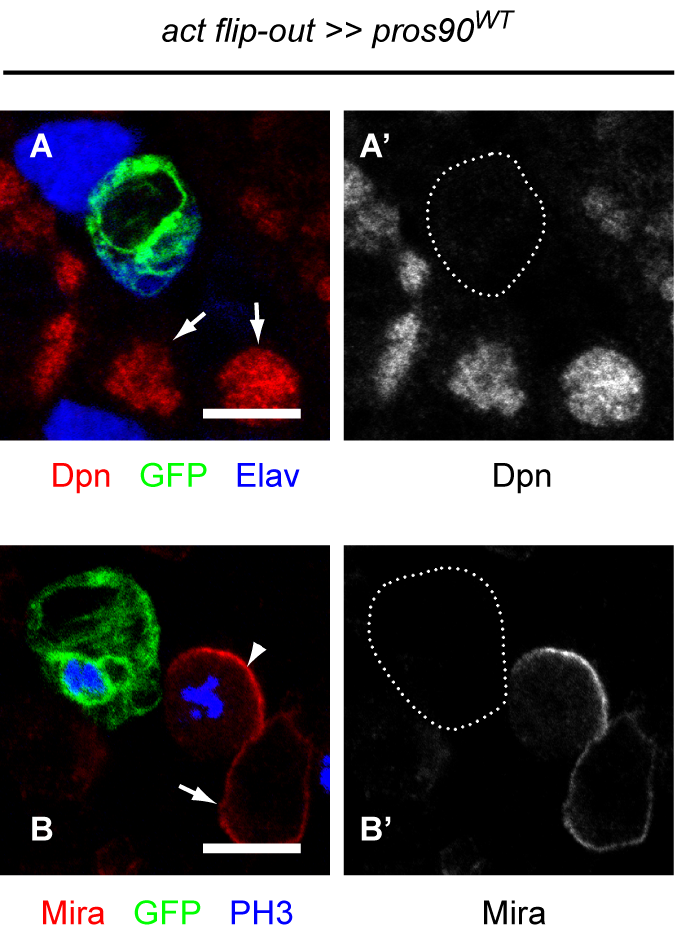

Supplement: Figure S2 — Mis-expression of pros abolished dpn expression and mislocalized Mira in the NBs. (A–A′) act-Gal4 driven pros expression in flip-out clones (marked by CD8:GFP in green) resulted in down-regulation of nuclear Dpn (red) in the NB, while neighbouring wt NBs exhibited strong nuclear Dpn (arrows) after 12 h of clonal induction. (B–B′) In the same clonal background, cortical Mira (red) was absent from an interphase NBs (as judged by the lacked of PH3 in blue), while two neighbouring NBs, in interphase (arrow) and metaphase (arrowhead), showed normal cortical enrichment of Mira. Scale bar = 10 µm. (TIF) [file pbio.1001494.s002.tif]

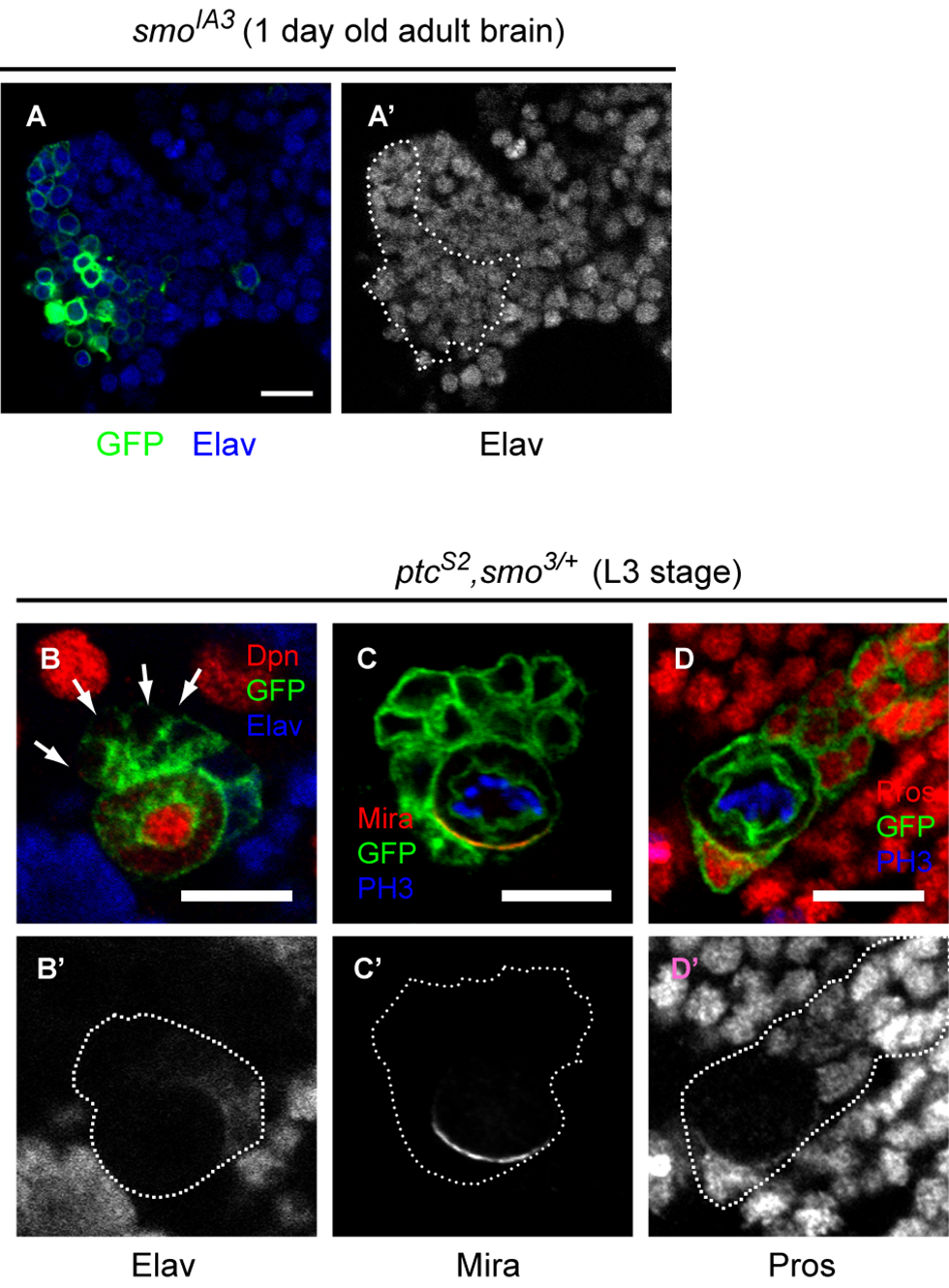

Supplement: Figure S3 — All cells in smoIA3 mutant clones expressed neuronal marker in adult brain. (A–A″) All the cells within smoIA3 clone (marked by CD8:GFP in green) in 1-d-old adult brain were Elav positive. (B–D′) MARCM clones for ptcS2; smo3/+ (marked by CD8:GFP in green) in late third instar larval brain. (B–B′) An example of a clone that contains four GMC-like cells (arrows) that were Dpn- (red) and Elav- (blue) negative. The mitotic NBs (as shown by the expression of PH3, blue) showed distinct Mira (red, C–C′) and Pros (red, D–D′) crescents. Scale bar = 10 µm. (TIF) [file pbio.1001494.s003.tif]

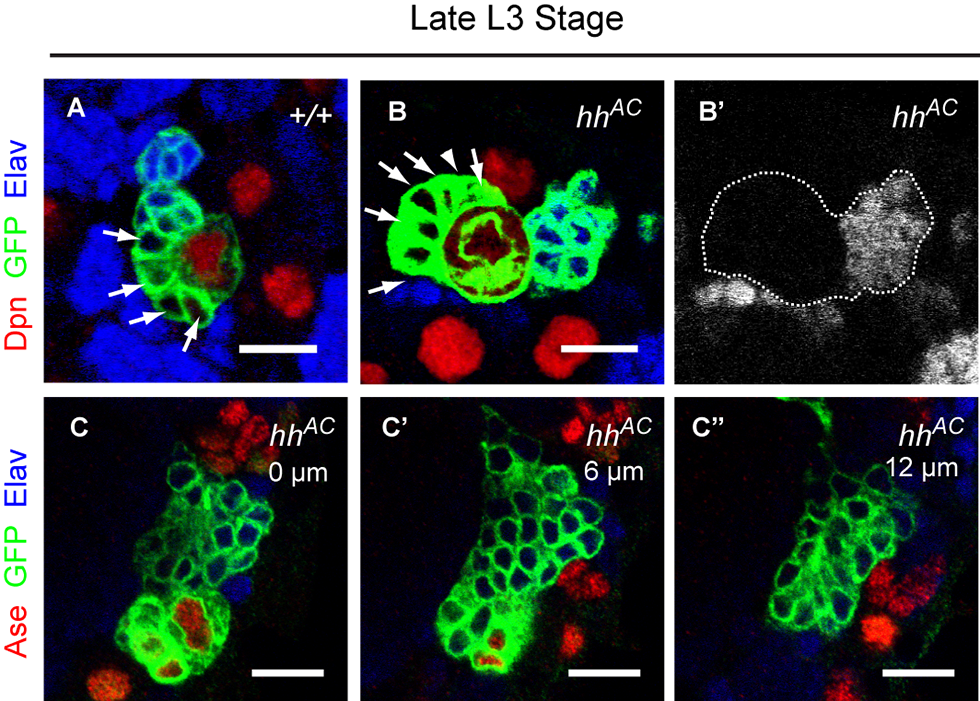

Supplement: Figure S4 — Hh ligand acted in a lineage restricted manner. (A) wt NB clone (marked by CD8:GFP, green) with four undifferentiated GMC-like cells, which were both Dpn- (red) and Elav- (blue) negative (arrows) as compared to (B–B′) hhAC clone, which showed six undifferentiated GMC-like cells (arrows; arrowhead marks one GMC that was partially hidden from view). (C–C″) Three consecutive z-sections (6 µm apart from each other) of a single hhAC clone. Scale bar = 10 µm. (TIF) [file pbio.1001494.s004.tif]

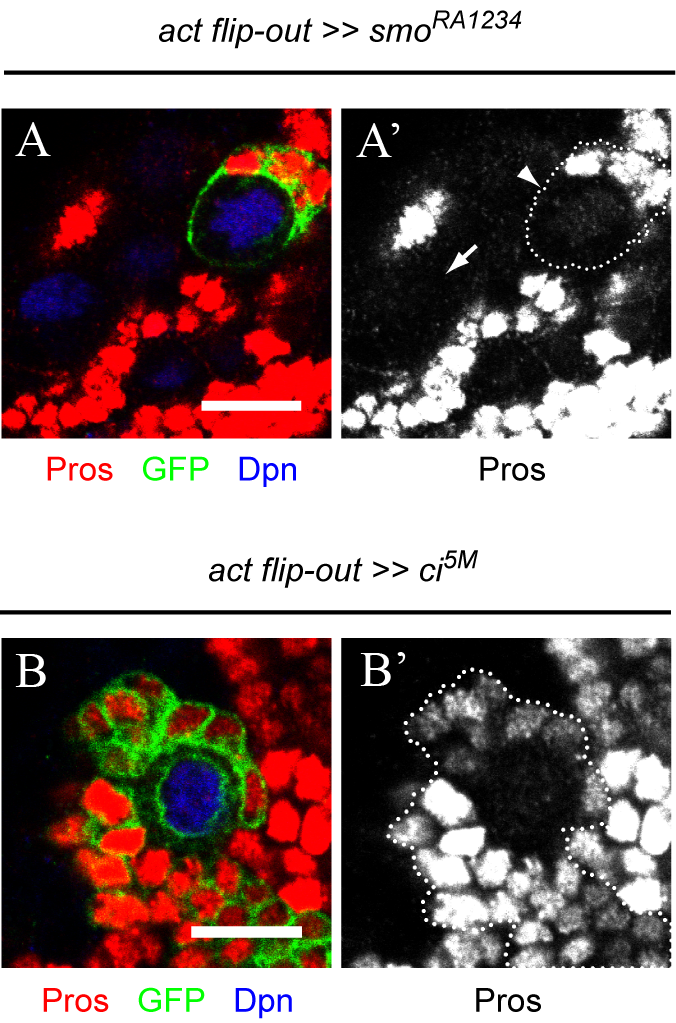

Supplement: Figure S5 — High levels of Hh signaling led to nuclear Pros localization in NBs. (A–B′) act-Gal4 flip-out driver induced clones (marked by CD8:GFP, green) that ectopically expressed smoRA1234 (A–A′) and ci5M (B–B′). The NBs (Dpn positive, blue) within the clones showed weak nuclear localization of Pros (red, arrowheads), while the neighbouring wt NB was devoid of nuclear Pros (arrow). Scale bar = 10 µm. (TIF) [file pbio.1001494.s005.tif]

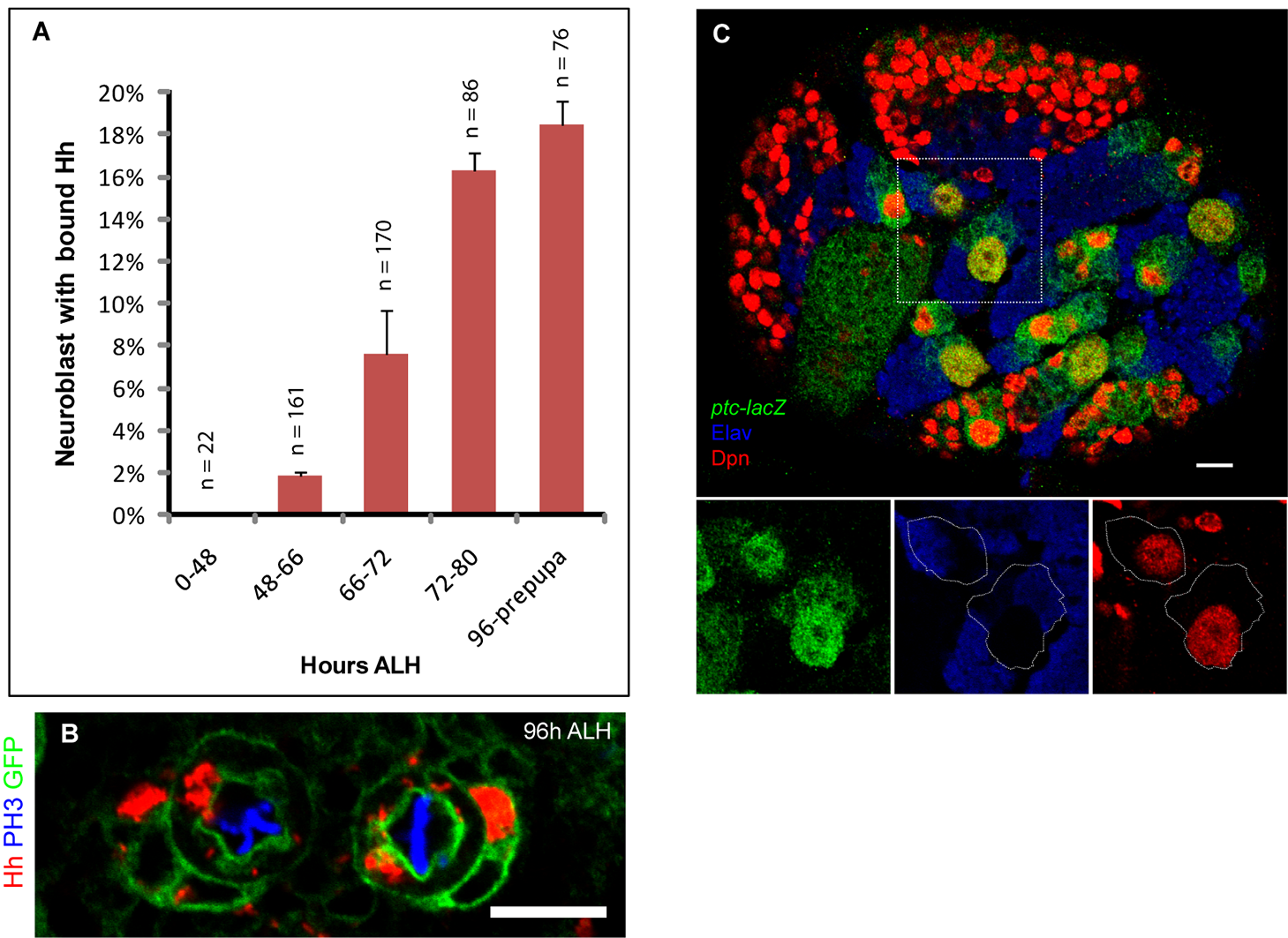

Supplement: Figure S6 — Hh signaling was perceived by the NBs. (A) The percentage of the NB with bound Hh was determined by calculating the number of NBs with bound Hh over the total number of NBs in the central brains of wt larvae at different age windows. Error bars corresponds to standard error of the mean (SEM). (B) Accumulation of Hh protein on/within the wt NBs (outlined by GFP, green) at 96 h ALH was visualized with anti-Hh antibody (red). (C) A wt third instar larval brain lobe was immunostained to show the expression of Dpn (red), Elav (blue), and the Hh reception reporter, ptc-lacZ (green). Inset showed two separate NB clones in which β-Gal expression was detected in the NB and, to a lesser extent, GMCs. Scale bar = 10 µm. (TIF) [file pbio.1001494.s006.tif]

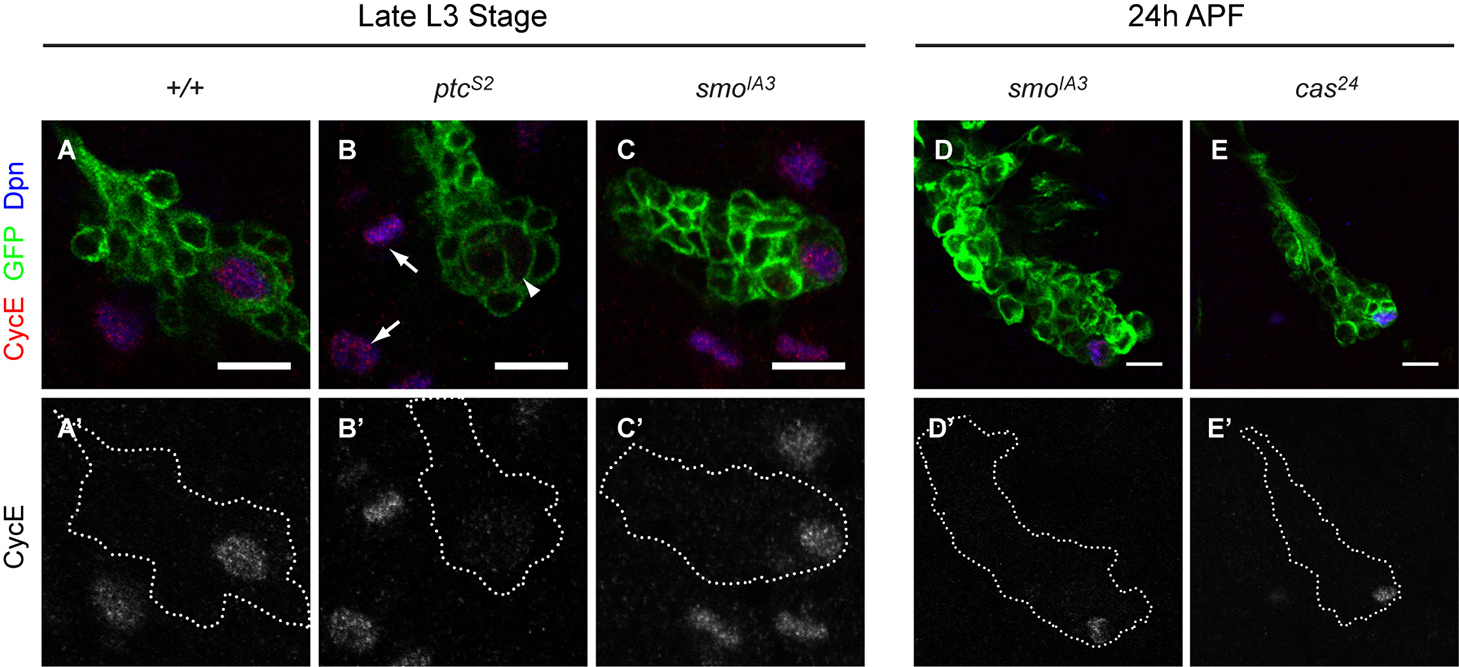

Supplement: Figure S7 — Proliferative status of the NB correlated with CycE expression. (A–C′) MARCM clones (marked by CD8:GFP, green) for wt (A–A′) and smoIA3 (C–C′) in late third instar larval brains contained a single Dpn- (blue) positive NB that co-expressed CycE (red). (B–B′) The expression of CycE was largely abolished in ptcS2 NB (arrowhead) as compared to the surrounding wt NBs outside the clone (arrows). (D–E′) At 24 h APF, smoIA3 (D–D′) and cas24 (E–E′) clones (marked by CD8:GFP, green) continued to express Dpn (blue) and CycE (red) when most of the surrounding wt NBs had already down-regulated both Dpn and CycE. Scale bar = 10 µm. (TIF) [file pbio.1001494.s007.tif]

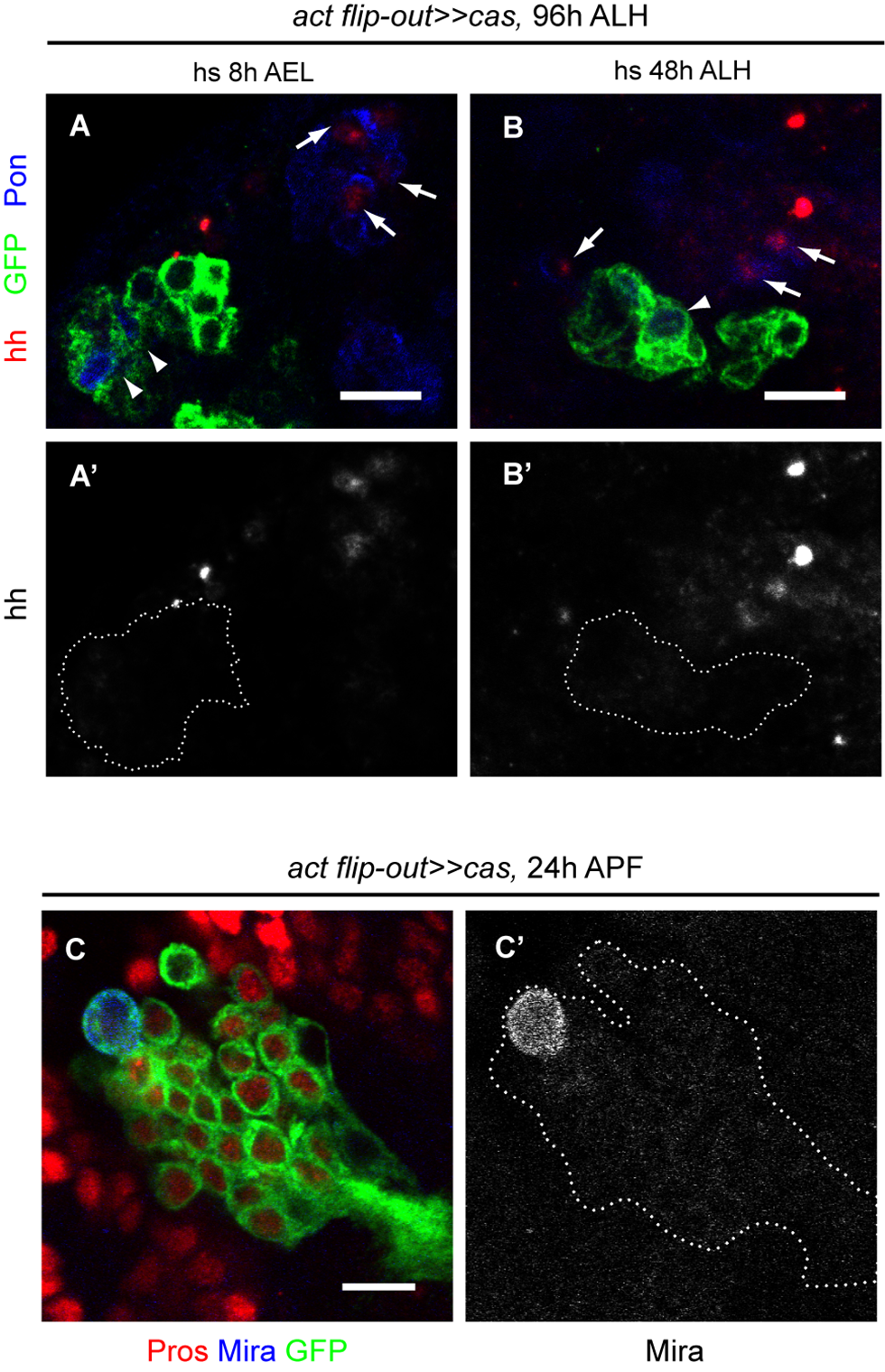

Supplement: Figure S8 — Mis-expression of cas crippled hh expression at 96 h ALH. (A–B′) In situ hybridization of hh mRNA (red) showed that mis-expression of cas in act-Gal4 flip-out clones (marked by CD8:GFP, green) affected hh expression when induced at both embryonic stage (A–A′) and late L2 stage (B–B′). Pon (blue) showed the outline of the newly born GMCs, which typically expressed hh mRNA. Note that the GMCs within the clones that mis-expressed cas (arrowheads) were devoid of hh transcript, while most of the surrounding wt GMCs (arrows) expressed hh normally. (C–C′) A clone that mis-expressed cas (CD8:GFP, green) continued to harbor a Mira-positive NB (blue) at 24 h APF. Scale bar = 10 µm. (TIF) [file pbio.1001494.s008.tif]

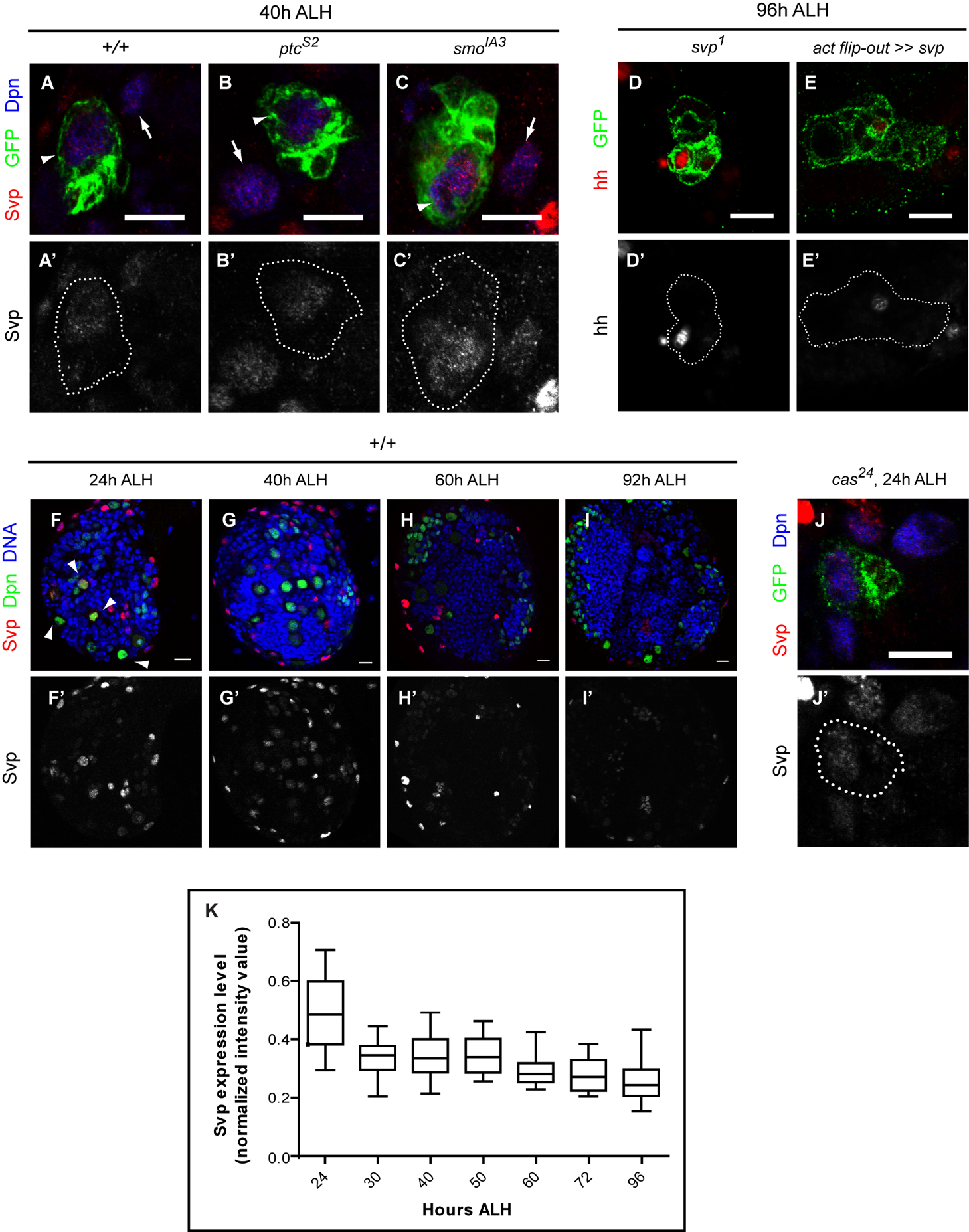

Supplement: Figure S9 — Hh signaling and Svp were unlikely to function in a linear pathway. (A–C′) NB clones at 40 h ALH for different genotype: wt (A–A′), ptcS2 (B–B′), and smoIA3 (C–C′) were marked by CD8:GFP in green. The NBs (labeled with Dpn, blue) within the clones (arrowheads) expressed Svp (red) in a manner that was indistinguishable from the neighbouring wt NBs (arrows). (D–E′) Both svp1 mutant (D–D′) and act-Gal4 flip-out driven svp mis-expression (E–E′) clones in the central brain (labeled by CD8:GFP, green) contained GMCs that expressed hh transcript (red) at 96 h ALH. (F–I′) The expression patterns of Svp in the brain lobe at various time points ALH. Svp was found to be expressed strongly in the NBs (co-labeled with Dpn, green) at 24 h (F–F′, arrowheads), and became progressively weaker as time passed: 40 h (G–G′), 60 h (H–H′), 92 h (I–I′). Svp was also found to be expressed in the neurons and glia that were non-Dpn positive. (J–J′) Embryonic clone of cas24 induced at 12–16 h AEL (marked by CD8:GFP, green) expressed Svp (red) in the NB, which was also expressing Dpn (blue). (K) A box-plot that showed the expression level of Svp in the NBs at different time points, normalized to the mean of the highest Svp expression in non-NB cells. The five number summaries were minimum, lower quartile, median, upper quartile, and maximum. Scale bar = 10 µm. (TIF) [file pbio.1001494.s009.tif]

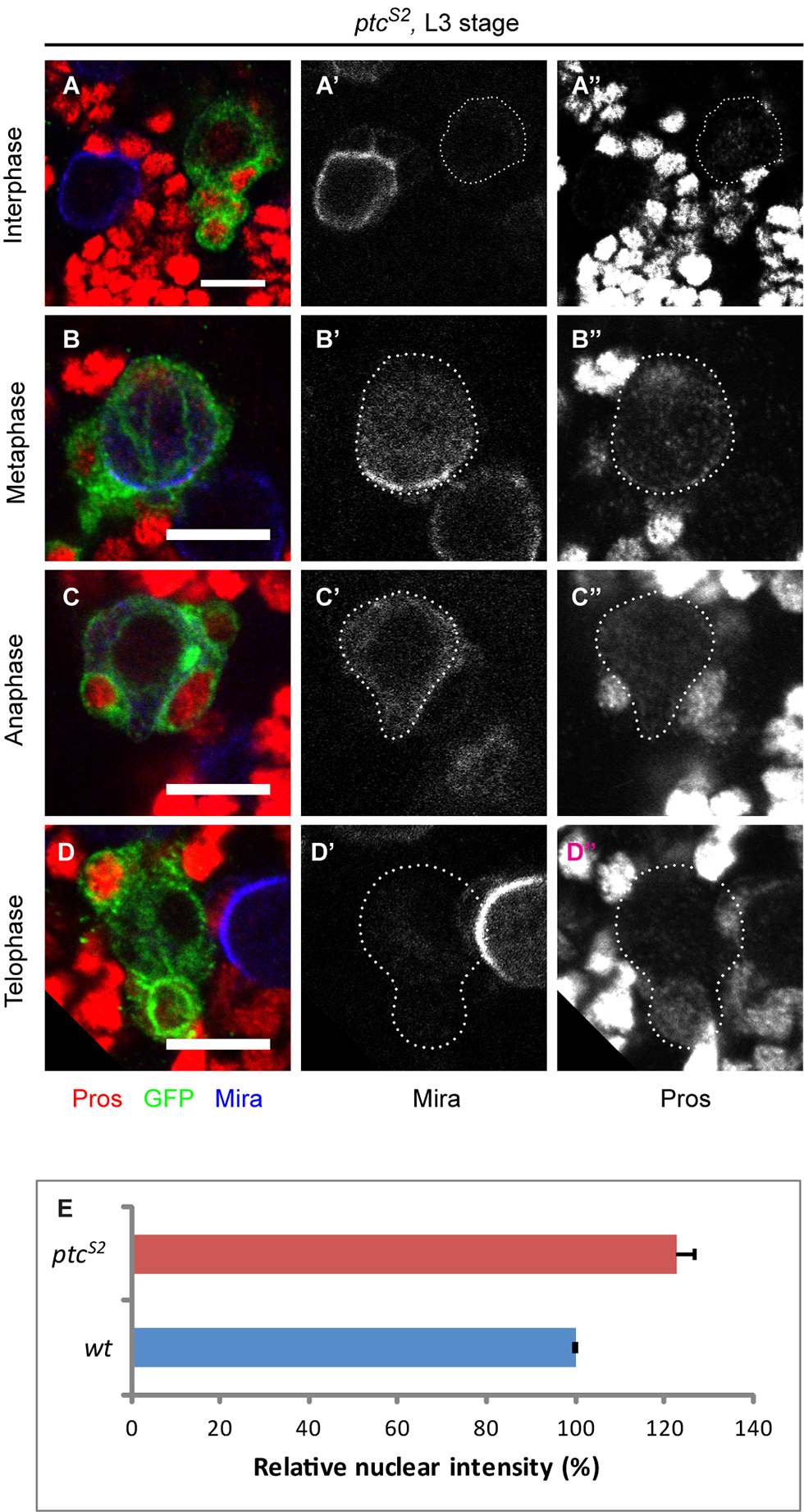

Supplement: Figure S10 — Mira and Pros failed to localize properly throughout the entire mitotic phase in ptcS2 NBs. (A–D″) ptcS2 NBs (marked by CD8:GFP, green) at different mitotic stages were examined for the localization of Mira (blue) and Pros (red). Interphase NBs (A–A″) were often devoid of cortical Mira and showed abnormal nuclear accumulation of Pros. Those NBs that managed to enter metaphase (B–B″) frequently showed weak Mira/Pros crescent, along with their cytoplasmic displacement that persisted through anaphase (C–C″) and telophase (D–D″), even though size asymmetry appeared to be unaffected. (E) Interphase ptcS2 NBs contained higher level of nuclear Pros, as measured by the intensity relative to neighbouring wt NBs. Error bars corresponds to standard deviation (SD). Scale bar = 10 µm. (TIF) [file pbio.1001494.s010.tif]

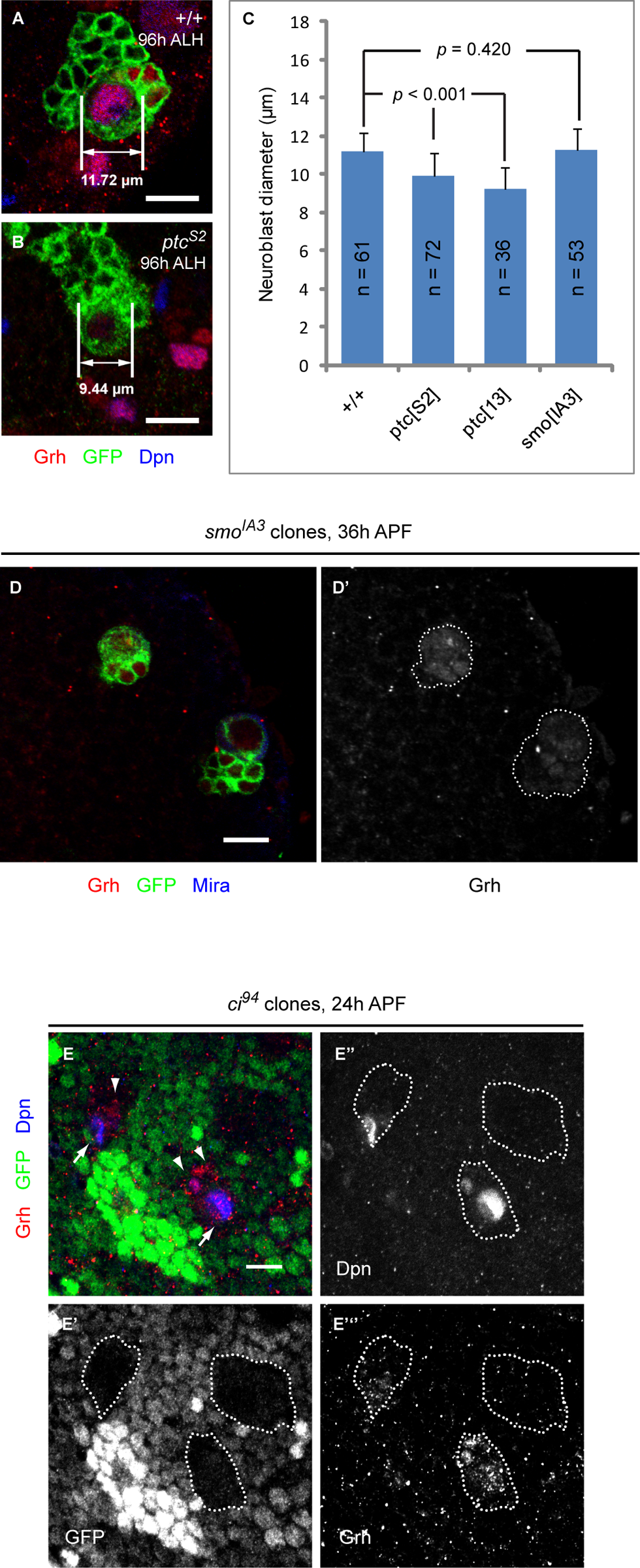

Supplement: Figure S11 — Hh signaling induced cell cycle exit in the NBs via down-regulation of Grh. (A–B) An example of NB size (diameter) in wt (A) and ptcS2 (B) clones at 96 h ALH. (C) Quantitation of NB diameter in wt, ptcS2, ptc13, and smoIA3 clones at 96 h ALH. Error bars represent standard deviation (SD) while statistical significance was determined using Student's t test. (D–D′) smoIA3 clones in the pupal brain (36 h APF) continue to express Grh (red) in NB and GMCs even though all other neighbouring wt cells had down-regulated the Grh expression. (E–E′″) ci94 clones, as marked by the absence of GFP (green, panel E′) at 24 h APF frequently contained NB that continued to express Dpn (blue) and Grh (red), as well as some GMCs which were Grh-positive as well. Scale bar = 10 µm. (TIF) [file pbio.1001494.s011.tif]

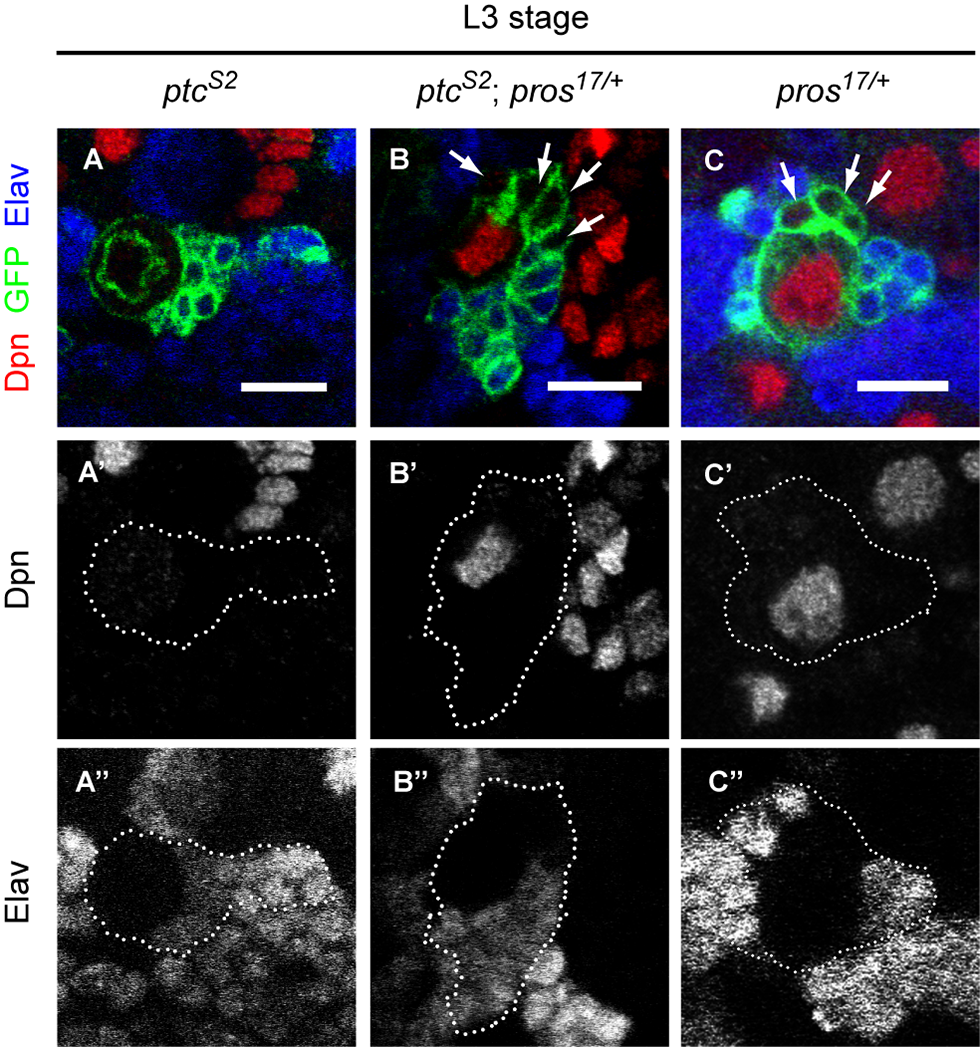

Supplement: Figure S12 — The phenotype of ptcS2 clones can be suppressed by removing one copy of pros . (A–A″) In ptcS2 clone (marked by GFP), all the cells other than the NB were expressing Elav (blue). (B–B″) The number of Dpn-negative, Elav-negative GMCs (arrows) was reverted to wt levels in homozygous ptcS2 clone when one copy of pros is removed. (C–C″) Removal of one copy of pros did not cause the expansion of GMC-like cells (arrow) by itself. Scale bar = 10 µm. (TIF) [file pbio.1001494.s012.tif]
