# Supplementary material for: Hedgehog Signaling Acts with the Temporal Cascade to Promote Neuroblast Cell Cycle Exit
Source: PLoS Biol. 2013 Feb 26;11(2):e1001494. doi: 10.1371/journal.pbio.1001494 (PMC3582610; doi:10.1371/journal.pbio.1001494)
Supplement: Text S1 — Supplemental material and methods. (DOC) [file pbio.1001494.s013.doc]

**Text S1:**

**Genotypes of the lines used**

Clonal generation

*Wildtype MARCM clone:* elav-GAL4, hsFLP, UAS-CD8::GFP/+; FRT40A/FRT40A tubP-GAL80

*ptc^S2^ MARCM clone*: elav-GAL4, hsFLP, UAS-CD8::GFP/+; FRT42D ptc^S2^/FRT42D tubP-GAL80

*smo^IA3^* MARCM clone: elav-GAL4, hsFLP, UAS-CD8::GFP /+; FRT40A smo^IA3^/FRT40A tubP-GAL80

*smo^IA3^; grh^RNAi^* MARCM clone: elav-GAL4, hsFLP, UAS-CD8::GFP /+; FRT40A smo^IA3^/FRT40A tubP-GAL80; P{GD9996}v33678/+

*cas^24^ MARCM clone*: elav-GAL4, hsFLP/+; UAS-nLacZ, UAS-CD8::GFP/+; FRT82B cas^24^/FRT82B tubP-GAL80

*svp^1^ MARCM clone*: elav-GAL4, hsFLP/+; UAS-nLacZ, UAS-CD8::GFP/+; FRT82B svp^1^/FRT82B tubP-GAL80

*flfl^795^ MARCM clone*: elav-GAL4, hsFLP/+; UAS-nLacZ, UAS-CD8::GFP/+; FRT82B flfl^795^/FRT82B tubP-GAL80

*ptc^RNAi^; cas^24^ MARCM clone*: elav-GAL4, hsFLP/+; UAS-nLacZ, UAS-CD8::GFP/ P{TRiP.JF03223}attP2; FRT82B cas^24^/FRT82B tubP-GAL80

*PP4-19C^RNAi^; cas^24^ MARCM clone*: elav-GAL4, hsFLP/+; UAS-nLacZ, UAS-CD8::GFP/ P{GD13525}v28795; FRT82B cas^24^/FRT82B tubP-GAL80

*ci^RNAi^; flfl^795^ MARCM clone*: elav-GAL4, hsFLP/+; UAS-nLacZ, UAS-CD8::GFP/ P{KK100760}v105620; FRT82B flfl^795^/FRT82B tubP-GAL80

*ptc^S2^; pros^17/+^ MARCM clone*: elav-GAL4, hsFLP, UAS-CD8::GFP/+; FRT42D ptc^S2^/FRT42D tubP-GAL80; pros^17^/+

*ptc^S2^; grh^o/e^ MARCM clone*: elav-GAL4, hsFLP, UAS-CD8::GFP/+; FRT42D ptc^S2^/FRT42D tubP-GAL80; UAS-*grh*/+

*ci^Nc5m5m^ flip-out clone*: hsFLP, UAS- GFP/+; Ay-GAL4/+; UAS-ci^Nc5m5m^/+

*smo^RA1234^ flip-out clone*: hsFLP, UAS- GFP/+; Ay-GAL4/+; UAS-smo^RA1234^/+

*ci^Nc5m5m^ flip-out clone*: hsFLP, UAS- GFP/+; Ay-GAL4/+; UAS-ci^Nc5m5m^/+

*ci^94^ clones*: y w hsp70-flp; FRT42 P[ci^+^] hsp70-GFP/FRT42; hh-lacZ/+; ci^94^/ci^94^

Live imaging

*Wildtype:* wor-GAL4, UAS-pon::GFP/Cyo; G147, UAS-histone::RFP/TM6B

*ptc^S2^ MARCM clones*: elav-GAL4, hsFLP, UAS-CD8::GFP/+; FRT42D ptc^S2^/FRT42D tubP-GAL80; UAS-histone2AvRFP/+

smo^IA3^ MARCM clones: elav-GAL4, hsFLP, UAS-CD8::GFP /+; FRT40A smo^IA3^/FRT40A tubP-GAL80; UAS-histone2AvRFP/+

Primer-pairs for qPCR

| hh-1 | Forward | TATGCCCCACAGAGGATATG |
| --- | --- | --- |
|  | Reverse | TGGTCGCCTTGGACAAAC |
| hh-2 | Forward | AGCTAAGCAAGCCGACAATG |
|  | Reverse | AATTTTTGAAGAAAAGCTGATCG |
| hh-3 | Forward | TCGGTGGATGTGAAGATGTG |
|  | Reverse | TCGGAGGCATGAATTTGC |
| hh-4 | Forward | ATCCCTGCTGCTTCTAATGC |
|  | Reverse | TAAATTGCGCGCGTTGTC |
| hh-5 | Forward | GAGTCGGAACGAAACGAAAC |
|  | Reverse | CAAACCATTCCGACCAACTG |
| hh-6 | Forward | AGGCCTAATGGAAACGAAGG |
|  | Reverse | TGCACACTTTAGTGGGTTGC |
| hh-7 | Forward | CGGCGCATAACAAAAAGG |
|  | Reverse | CTCAAGTGCTTAAGGGGTCTTAG |
| hh-8 | Forward | CACGGCTTAAATGCTGTTCC |
|  | Reverse | GCATGCGTTGATGGACTTG |
| hh-9 | Forward | TGAAGCTCGATGGGATTG |
|  | Reverse | TGAAGAGTATAGCCCACTTCGAC |
| hh-10 | Forward | CTGCCGACATTTGATTTGTG |
|  | Reverse | GAACAGGAAAGGCATTTTGG |
| hh-11 | Forward | ACATCGAGACACTTGGGATG |
|  | Reverse | ACCATAAAGGCACGAACAGC |
| hh-12 | Forward | CATAAGCCACATACCCACACC |
|  | Reverse | CTTACTTTTCGTTGGTGTTTCG |
| hh-13 | Forward | ACACATTGACGGGCGTATTC |
|  | Reverse | AACTTGGATCTGTGGCAAGC |
| hh-14 | Forward | GAAATTGCAGTTGCAGTCGAG |
|  | Reverse | GCCATGGATGAAAGTGTGTG |
| hh-15 | Forward | TGCTGCATCATCTGGTTGTC |
|  | Reverse | TATCGCCTCGAGTTCATTCC |
| hh-16 | Forward | TTCTTGAAAGCACCCTCGTC |
|  | Reverse | ACATTTCCGAGCGGAGTATC |
| hh-17 | Forward | CCAAATTCAAGGACCTCGTG |
|  | Reverse | ACGGTGTTTGAGGGTTTTCC |
| hh-18 | Forward | TGGGAAAGTTTCGCTTATCG |
|  | Reverse | TGGCGTAAGACTGGGTTTTC |
| hh-19 | Forward | TTGTCTGATAAGCGGACTGC |
|  | Reverse | GCGAACCCAATTTCTTTCAG |
| hh-20 | Forward | TCGGTGCCAGAACAAAATG |
|  | Reverse | ACCCACTCCACTTTCACTACC |
| hh-21 | Forward | AGCAGTTAGTGGGGTGGTTG |
|  | Reverse | AAGATAAGCTGGTGGGAAGC |
| hh-22 | Forward | ATCGCCCACACAAGTGTTAC |
|  | Reverse | TGAAACTCTAGCAGGCGAAC |
| hh-23 | Forward | CTTCGGGCGATTGACTAAAC |
|  | Reverse | TTCGGTAGCGGAACAGTTG |
| hh-24 | Forward | TGCAAGGAGCTGCAAAGAG |
|  | Reverse | CAGCTGAAATTCACGCACAG |
| hh-25 | Forward | GGCCGATAGCCGATATTTG |
|  | Reverse | GCTCGTCATCGCTTCATTAG |
| hh-26 | Forward | TCGAATCCTTTTCTGCTTGG |
|  | Reverse | CTTTGTTTGCCAATGTGGTG |
| hh-27 | Forward | TGATCCCACGGCTAATCTTG |
|  | Reverse | GATCGGAGATCAGCAATCTG |
| hh-28 | Forward | GGAGGTATTTTTAGCGAACCTG |
|  | Reverse | CCATGAAGGCGTCTATTTTTG |
| hh-29 | Forward | TGGACCACCATGTTTTACACC |
|  | Reverse | TTGGTGCCAACTGTTGTGAG |
| hh-30 | Forward | CACGTACTTCGCCTTGATCC |
|  | Reverse | TAATGATGGAGCTCGGCAAC |
| pdm-1 | Forward | TTTTATGGCCCACCAGAGAG |
|  | Reverse | GCCCATCGTTATGGAATCAC |
| pdm-2 | Forward | CTTCTCTGGGGTGTCAAATACC |
|  | Reverse | AATTGCGTTGTCTGCCTACC |
| pdm-3 | Forward | TTTATGGACGCGCACAGTAG |
|  | Reverse | AGCAGTGCCTCTAGGGTATCAC |
| pdm-4 | Forward | GTGGAAATCGAGGCAGTTTG |
|  | Reverse | TTCGGCCCAGTATGGTTTAG |
| act-5C | Forward | TCACATGCCGCAACTGATAG |
|  | Reverse | GAGAATTTCCTCCGCAACTG |
